# Prevalence and Predictability of Low-Yield Inpatient Laboratory Diagnostic Tests

**DOI:** 10.1001/jamanetworkopen.2019.10967

**Published:** 2019-09-11

**Authors:** Song Xu, Jason Hom, Santhosh Balasubramanian, Lee F. Schroeder, Nader Najafi, Shivaal Roy, Jonathan H. Chen

**Affiliations:** 1 Center for Biomedical Informatics Research, Department of Medicine, Stanford University, Stanford, California; 2 Division of Hospital Medicine, Department of Medicine, Stanford University, Stanford, California; 3 Department of Pathology, University of Michigan School of Medicine, Ann Arbor; 4 Department of Medicine, University of California, San Francisco; 5 Department of Computer Science, Stanford University, Stanford, California

## Abstract

**Importance:**

Laboratory testing is an important target for high-value care initiatives, constituting the highest volume of medical procedures. Prior studies have found that up to half of all inpatient laboratory tests may be medically unnecessary, but a systematic method to identify these unnecessary tests in individual cases is lacking.

**Objective:**

To systematically identify low-yield inpatient laboratory testing through personalized predictions.

**Design, Setting, and Participants:**

In this retrospective diagnostic study with multivariable prediction models, 116 637 inpatients treated at Stanford University Hospital from January 1, 2008, to December 31, 2017, a total of 60 929 inpatients treated at University of Michigan from January 1, 2015, to December 31, 2018, and 13 940 inpatients treated at the University of California, San Francisco from January 1 to December 31, 2018, were assessed.

**Main Outcomes and Measures:**

Diagnostic accuracy measures, including sensitivity, specificity, negative predictive values (NPVs), positive predictive values (PPVs), and area under the receiver operating characteristic curve (AUROC), of machine learning models when predicting whether inpatient laboratory tests yield a normal result as defined by local laboratory reference ranges.

**Results:**

In the recent data sets (July 1, 2014, to June 30, 2017) from Stanford University Hospital (including 22 664 female inpatients with a mean [SD] age of 58.8 [19.0] years and 22 016 male inpatients with a mean [SD] age of 59.0 [18.1] years), among the top 20 highest-volume tests, 792 397 were repeats of orders within 24 hours, including tests that are physiologically unlikely to yield new information that quickly (eg, white blood cell differential, glycated hemoglobin, and serum albumin level). The best-performing machine learning models predicted normal results with an AUROC of 0.90 or greater for 12 stand-alone laboratory tests (eg, sodium AUROC, 0.92 [95% CI, 0.91-0.93]; sensitivity, 98%; specificity, 35%; PPV, 66%; NPV, 93%; lactate dehydrogenase AUROC, 0.93 [95% CI, 0.93-0.94]; sensitivity, 96%; specificity, 65%; PPV, 71%; NPV, 95%; and troponin I AUROC, 0.92 [95% CI, 0.91-0.93]; sensitivity, 88%; specificity, 79%; PPV, 67%; NPV, 93%) and 10 common laboratory test components (eg, hemoglobin AUROC, 0.94 [95% CI, 0.92-0.95]; sensitivity, 99%; specificity, 17%; PPV, 90%; NPV, 81%; creatinine AUROC, 0.96 [95% CI, 0.96-0.97]; sensitivity, 93%; specificity, 83%; PPV, 79%; NPV, 94%; and urea nitrogen AUROC, 0.95 [95% CI, 0.94, 0.96]; sensitivity, 87%; specificity, 89%; PPV, 77%; NPV 94%).

**Conclusions and Relevance:**

The findings suggest that low-yield diagnostic testing is common and can be systematically identified through data-driven methods and patient context–aware predictions. Implementing machine learning models appear to be able to quantify the level of uncertainty and expected information gained from diagnostic tests explicitly, with the potential to encourage useful testing and discourage low-value testing that incurs direct costs and indirect harms.

## Key Points

**Question:**

How prevalent are low-yield inpatient diagnostic laboratory tests for which results are predictable with machine learning models?

**Findings:**

In this diagnostic study of 191 506 inpatients from 3 tertiary academic medical centers, common low-yield inpatient diagnostic laboratory test results were systematically identified through data-driven methods and personalized predictions.

**Meaning:**

The findings suggest that data-driven methods can make explicit the level of uncertainty and expected information gain from diagnostic tests, with the potential to encourage useful testing and discourage low-value testing that can incur direct cost and indirect harm.

## Teaser

This diagnostic study uses machine learning models to assess the prevalence of low-yield inpatient laboratory tests.

## Introduction

Unsustainable growth in health care costs is exacerbated by waste that does not improve health.^1,2^ The Institute of Medicine estimates that more than $200 billion a year is spent on unnecessary tests and procedures.^3^ Given this amount of misallocated resources, there has been an increasing emphasis on high-value care, notably with the American Board of Internal Medicine Foundation’s Choosing Wisely guidelines.^4^ Laboratory testing, in particular, constitutes the highest-volume medical procedure,^5^ with estimates of up to 25% to 50% of all inpatient testing being medically unnecessary.^6,7^ The consequences of unnecessary testing are not simply financial but also include low patient satisfaction, sleep fragmentation, risk of delirium, iatrogenic anemia, and increased mortality.^8,9,10,11^

Numerous interventions have been studied to reduce inappropriate laboratory testing, including clinical education, audit feedback, financial incentives, and electronic medical record (EMR)–based ordering restrictions.^12,13,14,15^ Interventions based on EMRs offer pertinent information for clinical decision-making, such as cost, turnaround time, prior stable results, and guideline-based best practice alerts.^16,17,18,19,20^ Despite these efforts, unnecessary tests remain prolific when practitioners are influenced by fear of missing problems, medicolegal concerns, patient preferences, and the overall difficulty of systematically identifying low-value testing at the point of care, prompting behavior to check just in case.^21,22^

We envisioned patient-specific estimates of the pretest probability of results for any diagnostic test, displayed at the point of clinical order entry. When humans tend to have poor intuition for estimating probabilities and diagnostic test performance, having automated computer systems explicitly provide those estimates could substantially change clinical practice.^23^ Machine learning in medicine now offers a direct mechanism to produce such estimates by predicting select laboratory results.^24,25,26,27,28,29,30^ Although prior approaches can provide a laboratory result given other simultaneously available results (eg, estimating ferritin levels when other components of an iron panel are given), this is too late for decision support to change behavior when the tests are already performed. We addressed the more clinically relevant question of predicting laboratory results with only information available before the test is ordered.

Our objective was to identify inpatient diagnostic laboratory testing with predictable results that are unlikely to yield new information. Our analytic approach escalated from descriptive statistics to machine learning models for individualized estimates of predictable test results.

## Methods

This diagnostic study followed the Transparent Reporting of a Multivariable Prediction Model for Individual Prognosis or Diagnosis (TRIPOD) reporting guideline for reporting results of multivariate prediction models^31^ to develop and evaluate our machine learning methods (eFigure 1 in the Supplement gives an overview of our approach). Ten years (January 1, 2008, to December 31, 2017) of inpatient electronic medical record (EMR) data from hospitals at Stanford University, 4 years (January 1, 2015, to December 31, 2018) of data from University of Michigan (UMich), and 1 year (2018) of data from University of California, San Francisco (UCSF) were used for this study. To preserve data privacy, raw clinical data were deidentified, processed, trained, and evaluated locally at each local site, with only evaluation results sent back to Stanford for further analysis. The Stanford University, UMich, and UCSF institutional review boards approved the study at each site. Project-specific informed consent was not required because the study was restricted to secondary analysis of existing clinical data. Patient data at Stanford University were extracted and deidentified by the STRIDE (Stanford Translational Research Integrated Database Environment) project, a research and development project at Stanford University to create a standards-based informatics platform supporting clinical and translational research.

### Participants and Inclusion Criteria

All laboratory test results had reference labels for normal vs abnormal results as defined by local clinical laboratory reference ranges and at least 500 occurrences in a data set. For each laboratory test, we retrieved a random sample of 10 000 test orders from the available data (or all orders if <10 000).

### Outcome and Evaluation Metrics

Our goal was to predict the result (negative vs positive) of each laboratory test using information available before the order was placed. We considered stand-alone tests, in which a single order yielded a single result (eg, magnesium level, lactate level, or blood cultures), and panel tests that yielded multiple component results (eg, a complete blood cell count panel yielded white blood cell, hemoglobin, and platelet component results). We predicted the results of each panel component separately to avoid labeling an entire panel as positive or negative. We evaluated prediction performance through standard metrics for diagnostic accuracy, including the area under the receiver operating characteristic curve (ie, AUROC or C statistic), which summarizes the trade-off between sensitivity and specificity.^32^ Given specific decision thresholds, we calculated diagnostic test metrics, including sensitivity, specificity, positive predictive value, and negative predictive value (NPV). Typically, such metrics evaluate how well a test predicts a diagnosis. In our case, a test result being abnormal was itself the diagnosis, whereas the prediction algorithms operated as screening tests compared with the physical laboratory tests. For example, NPV was the probability of being correct when a negative or normal result was predicted.

### Predictors and Data Feature

For each laboratory, 875 raw features from the Stanford University EMR that reflected patient clinical context available at the time of the order entry were extracted (eTable 1 in the Supplement). The core features included patient demographics, normality of the most recent test of interest, numbers of recent tests of interest, history of Charlson Comorbidity Index categories, which specialty team was treating the patient, time since admission, time of day and year of the test, and summary statistics of recent vital statistics and laboratory results. Vital statistics and treatment team information were not accessible in the UMich data sample, which yielded 603 raw features. Age and sex information were not accessible in the UCSF data sample, which yielded 806 raw features.

### Development vs Validation Split

Patients were randomly split into training (development) and held-out test (validation) sets with a 75:25 split. The model was developed based on the training data alone but assessed generalizable predictive accuracy on the separate patients in the held-out sets.

### Missing Data

Most of the data features, such as history of a comorbidity category or the number of prior laboratory tests, always had a valid value (including not present or zero). Numerical results (eg, mean sodium level in the past week) could be missing, in which case we carried forward the most recent value from the patient’s prior records. If no prior values existed, we imputed the training sample mean.

### Feature Selection

We applied recursive feature elimination (with cross-validation) to select the top 5% most important features for model building that best improved accuracy when included in prediction models. This resulted in 43 processed features in each subsequent prediction model (the eMethods in the Supplement gives technical explanations).

### Model Development

**Figure 1.**
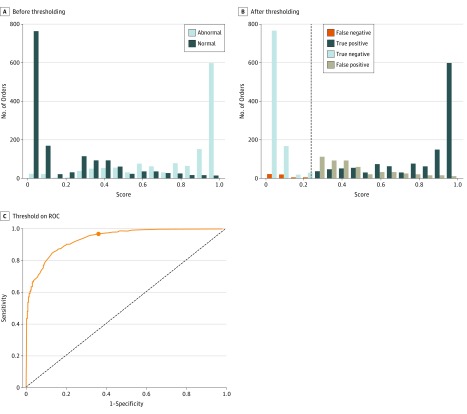
Normality Scores, Decision Threshold, and Receiver Operating Characteristic (ROC) Curve A, Histogram of normality scores distributed among normal and abnormal orders. B, After picking a threshold, orders were classified as predicted to be normal if their normality scores were above the threshold or predicted to be abnormal if they were below the threshold. True-negative orders were predicted to be normal but actually were normal, false-negative orders were predicted to be normal but actually were abnormal, false-positive orders were predicted to be abnormal but actually were normal, and true-positive orders were predicted to be abnormal but actually were abnormal. C, This choice of threshold led to a sensitivity of 96% and specificity of 67%, as shown on the ROC curve.

We built an array of prediction models using established algorithms,^33^ including regularized logistic regression, regress and round, naive Bayes, neural network multilayer perceptrons, decision tree, random forest, AdaBoost, and XGBoost.^34^ Each model generated a prediction score between 0 and 1 for how likely a laboratory test result would be negative or normal vs positive or abnormal (Figure 1A). A baseline model predicted the most recent result (if the patient had a prior test) or the overall prevalence of positive results as the prediction score. Additional model specifications are included in eTable 2 in the Supplement.

### Decision Threshold Estimation

Decision thresholds translate continuous prediction scores into discrete negative vs positive predictions (Figure 1B). We conservatively favored high sensitivity and high NPV to minimize the risks of alert fatigue and missing clinically important laboratory test result abnormalities (Figure 1C).^35,36^ This article gives results targeting an NPV of 95%, recognizing the diminishing returns of expected information gain when one is already 95% certain of the result.^37^ These diminishing returns were easily adjusted for different clinical scenarios with varying tolerances for uncertainty because we confirmed robustness across a range of options targeting NPVs of 99%, 95%, 90%, and 80% (eTable 3 in the Supplement).

### Statistical Analysis

To assess the statistical significance of the results, we calculated 95% CIs for AUROCs by resampling the evaluation set 1000 times for each laboratory (Table and eTables 3-8 in the Supplement). We performed additional randomized permutation tests to compare the AUROC of the best-performing algorithm against that of the baseline model (eFigures 2-7 in the Supplement).

**Table.** Diagnostic Performance Metrics of Top-Volume Stand-Alone Laboratory Tests Predicting Whether Laboratory Tests Will Yield a Normal Result on a Held-Out Evaluation Set, Targeting at an NPV of 95%

Laboratory Test	No. of Orders/1000 Patient Encounters	AUROC (95% CI)	Metric, %								
			Prevalence^a^	NPV	PPV	Sensitivity	Specificity	TN	FN	TP	FP
Magnesium	4246	0.76 (0.74-0.78)	26	91	36	86	47	35	3.6	22	39
Prothrombin time	2244	0.89 (0.88-0.91)	80	85	81	100	3.6	0.7	0.1	80	19
Phosphorus	2120	0.74 (0.72-0.76)	33	88	39	91	30	20	2.8	30	47
Partial thromboplastin time	1471	0.86 (0.85-0.87)	61	87	65	98	17	6.5	1.0	60	32
Lactate	1230	0.87 (0.85-0.88)	29	91	56	82	74	53	5.2	23	19
Calcium, ionized	1197	0.72 (0.70-0.74)	61	90	62	100	4.8	1.9	0.2	61	37
Potassium	752	0.81 (0.79-0.84)	12	92	40	43	91	80	7.0	5.2	7.9
Troponin I	534	0.92 (0.91-0.93)	33	93	67	88	79	53	4.0	29	14
LDH	455	0.93 (0.93-0.94)	47	95	71	96	65	35	1.8	45	18
Blood culture											
Aerobic and anaerobic	400	0.66 (0.61-0.71)	8.1	93	16	16	93	85	6.8	1.3	6.6
2 Aerobic	371	0.62 (0.58-0.67)	9.1	93	12	61	54	49	3.6	5.6	42
Sodium	361	0.92 (0.91-0.93)	57	93	66	98	35	15	1.1	56	28

Abbreviations: AUROC, area under the receiver operating characteristic curve; FN, false-negative; FP, false-positive; LDH, lactate dehydrogenase; NPV, negative predictive value; PPV, positive predictive value; TN, true-negative; TP, true-positive.

^a^ Prevalence of abnormal or positive test results.

### Multisite Evaluation

We performed equivalent analysis from multiple sites, including hospitals at Stanford University, UMich, and UCSF. We developed mapping software between the data formats from different sites to allow for a common analytic process at each site without sharing raw clinical data. We cross-evaluated performances of models trained at one site and then tested at another.

## Results

### Prevalence of Repetitive Tests

**Figure 2.**
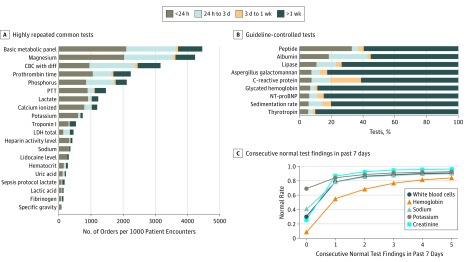
Prevalence of Repetitive Tests and Their Diminishing Information Gain A, Most commonly repeated laboratory test orders from July 1, 2014, to June 30, 2017, at Stanford University Hospital. The total length of each bar represents the total volume of laboratory orders per 1000 patient encounters, with shaded regions reflecting how many of these were repeated orders within a given time. For example, 47% of basic metabolic panels were subsequent tests performed again within 24 hours of the past order. Results were sorted by this number of repeated tests within 24 hours. B, Distribution of repeated orders for laboratory tests specifically identified as rarely ever having clinical justification for repeated daily testing.^38^ For example, 18.3% of albumin and 6.7% of glycated hemoglobin inpatient tests were performed again within 24 hours, even when it was not biologically plausible for the results to meaningfully change that rapidly. C, Prevalence of normal results for common laboratory components progressively increased toward 100% as more subsequent normal results were observed in the prior week. CBC with diff indicates complete blood cell count with differential; LDH, lactate dehydrogenase; NT-proBNP, N-terminal pro–brain-type natriuretic peptide; and PTT, partial thromboplastin time.

The recent data sets (July 1, 2014, to June 30, 2017) from Stanford University Hospital included 22 664 female inpatients (mean [SD] age, 58.8 [19.0] years) and 22 016 male inpatients (mean [SD] age, 59.0 [18.1] years). Figure 2A reports the overall volume of the most commonly repeated inpatient laboratory tests at Stanford University during that period. Among the top 20 volume tests, 792 397 were repeats of orders within 24 hours. Figure 2B reports the repetition rate of common tests medically implausible to yield new information from frequent testing (eg, glycated hemoglobin).^38^ The likelihood of common laboratory components that yielded a negative result progressively increased as repeated negative results were observed (Figure 2C).

### Model Performance

**Figure 3.**
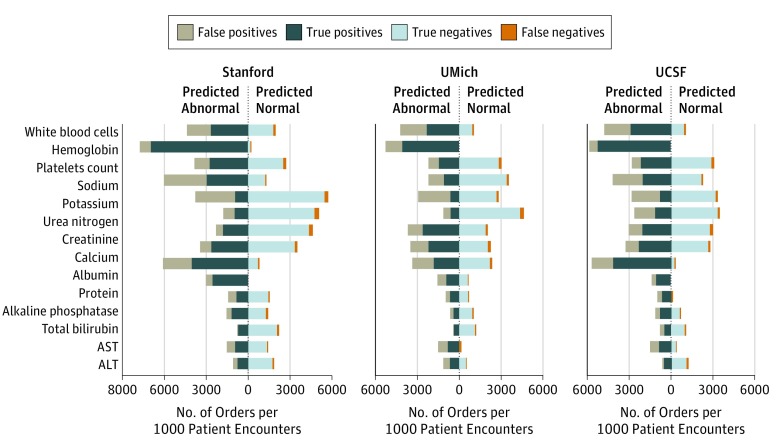
Diagnostic Metrics of Predictions on Common Components in the Data Sets of Stanford University, University of Michigan (UMich), and University of California, San Francisco (UCSF) Fractions of true-negative, false-negative, false-positive, and true-positive results are scaled by the number of orders among each 1000 patient encounters. Predicted normal represents the volume that the model would suggest not to order, and we targeted to limit the fraction of false-negative results to less than 5%. For some laboratory tests (eg, albumin measurement at Stanford University), there were almost zero predicted normal results, which means that a few orders existed in the training set that were unpredictable; thus, the predictor could not confidently achieve a 95% negative predictive value by picking any threshold above 0. The model chose a decision threshold equal to 0, which led to scores of all orders in the test set falling above the decision threshold, thus always encouraging ordering the test. ALT indicates alanine aminotransferase; AST, aspartate aminotransferase.

Random forest and XGBoost demonstrated the highest discriminating power for most of the stand-alone laboratory tests from Stanford University hospital, yielding a mean AUROC of 0.77 compared with 0.67 with the baseline model (eFigure 2 in the Supplement gives the ROC curves). The best-performing machine learning models predicted normal results with an AUROC of 0.90 or greater for 12 stand-alone laboratory tests (eg, sodium AUROC, 0.92 [95% CI, 0.91-0.93]; sensitivity, 98%; specificity, 35%; PPV, 66%; NPV, 93%; lactate dehydrogenase AUROC, 0.93 [95% CI, 0.93-0.94]; sensitivity, 96%; specificity, 65%; PPV, 71%; NPV, 95%; and troponin I AUROC, 0.92 [95% CI, 0.91-0.93]; sensitivity, 88%; specificity, 79%; PPV, 67%; NPV, 93%) and 10 common laboratory test components (eg, hemoglobin AUROC, 0.94 [95% CI, 0.92-0.95]; sensitivity, 99%; specificity, 17%; PPV, 90%; NPV, 81%; creatinine AUROC, 0.96 [95% CI, 0.96-0.97]; sensitivity, 93%; specificity, 83%; PPV, 79%; NPV, 94%; and urea nitrogen AUROC, 0.95 [95% CI, 0.94, 0.96]; sensitivity, 87%; specificity, 89%; PPV, 77%; NPV 94%). Diagnostic performance metrics for the most common stand-alone laboratory tests when targeting 95% NPV are given in the Table, with the full table of all laboratory tests evaluated in eTable 3 in the Supplement. Performance metrics for the common components in complete blood cell counts and comprehensive metabolic panels are given in Figure 3 along with results from UMich and UCSF data, with the full table of diagnostic performance metrics in eTable 4 in the Supplement and ROC curves in eFigure 3 in the Supplement.

### Model Transferability

**Figure 4.**
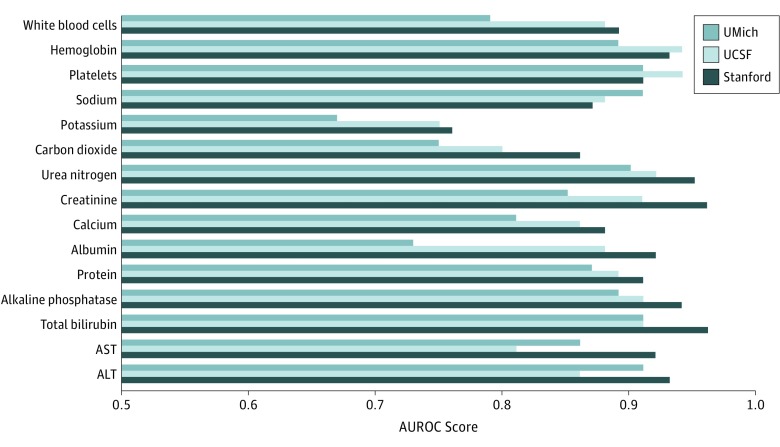
Area Under the Receiver Operating Characteristic Curve (AUROC) Scores of Models for 15 Common Laboratory Test Components Developed at Stanford University but Evaluated at All Sites The model generally achieved highest performance when evaluated locally at Stanford University with an AUROC of 0.9 or greater for 10 laboratory test components but still retained at 0.85 or greater in 9 cases when evaluated remotely at University of California, San Francisco (UCSF) and University of Michigan (UMich).

The respective prediction results for UMich data are reported in eFigure 4, eFigure 5, eTable 5, and eTable 6 in the Supplement, whereas similar results from UCSF are reported in eFigure 6, eFigure 7, eTable 7, and eTable 8 in the Supplement. Figure 4 gives the performance of models trained at Stanford University and subsequently evaluated at all sites. Although cross-site performance declined compared with local performance (eg, when predicting albumin results, AUROC decreased from 0.92 [95% CI, 0.91-0.94] when locally tested at Stanford University to 0.73 [95% CI, 0.70-0.75] when remotely tested at UMich), predictive power was retained (AUROC, >0.85) for most laboratory components (eTable 9 in the Supplement gives the full comparison data). For certain tests, such as sodium level, however, the model trained at Stanford University had a better AUROC when tested at UMich (0.91; 95% CI, 0.90-0.93) than locally at Stanford University (0.87; 95% CI, 0.85-0.88). Inspection of the data and model showed that the UMich sodium level was easier to predict, with a baseline model already yielding an AUROC of 0.87 at UMich and 0.79 at Stanford University.

## Discussion

### Interpretation

This study systematically identified low-yield diagnostic laboratory tests. Starting with simple descriptive statistics, Figure 2 shows how frequently laboratory tests are performed again. Although some tests may have credible reasons for such frequent repetition, guidelines and external knowledge can help identify some low-value repeated tests.^38^ For example, hundreds of tests for serum albumin, thyrotropin, and glycated hemoglobin levels were performed again within 24 hours, along with tens of thousands of repetitive tests for phosphorus and complete blood cell counts with differential. This finding quantitatively supports issues suggested in previous guidelines that hospitals can immediately use to target unnecessary repeated tests, such as through best practice alerts showing recently available test results.^12,19,38,39,40^

Most instances of low-yield testing are not as straightforward to identify; thus, our study added machine learning methods for personalized test result predictions. Additional features, such as patient demographics, vital signs, and other common laboratory results, can be synthesized through machine learning models to produce more robust and accurate predictions. Although different applications and clinical contexts will have different tolerances for uncertainty, the study gave the primary results when choosing a conservative target NPV close to 95% (when the model predicted a test result was going to be normal, the goal was for it to be correct 95% of the time). This approach fits a scenario in which these targets are implemented as best practice alerts with a desire to maintain a small number of false-positive results (5%). The results at this level of pretest were estimated by which pursuing further testing would yield markedly diminishing returns.^37^ eTables 3 through 8 in the Supplement give similar results across a range of different NPV targets.

Consistent with existing guideline-based forms of clinical decision support, pretest estimates of whether a laboratory test result will be normal would inform physician decision-making but not dictate or replace it. Ultimately, medical testing decisions are always based on varying levels of diagnostic certainty,^41^ even if practitioners are only implicitly aware that they are empirically estimating probabilistic risks based on patient characteristics. For example, blood cultures are not performed for every febrile patient because a credible risk of bacteremia is qualitatively recognized in only certain situations. Likewise, blood cultures are performed in sets of 4 bottles at a time, but we do not continue to check 5 or more bottles because we recognize further repeated tests are unlikely to yield information that was not already predictable based on the prior results. This approach provides a systematic and quantitative way to inform such decisions. The results should encourage practitioners and quality improvement committees to make explicit and quantitative their own embedded assumptions on acceptable decision thresholds. The general framework presented to quantify uncertainty can then feed into individual point-of-care decisions or more formal decision analyses.^42^

### Implications

This study provides a general approach to identifying predictable laboratory tests. Many of the laboratory tests that we evaluated have been evaluated for overuse, including magnesium level,^15,43,44,45^ blood cultures,^46^ and complete blood cell counts.^47^ Patient-specific estimates of laboratory test result normality at the point-of-order entry may discourage low-yield tests with predictably negative results and encourage appropriate tests with high levels of uncertainty. For example, when our method did not predict a blood culture result to be negative, this corresponds to greater than 16% positive predictive value (Table). This finding is more than enough risk of bacteremia to prompt diagnostic testing and even empirical treatment.

This approach can also raise questions on how guideline- and protocol-based testing is implemented and could be optimized. The optimal threshold of acceptable uncertainty depends on the clinical scenario and the particular test. For example, although screening tests (eg, HIV testing or pregnancy screens in hospital settings) have predictable normal results, most of the time, they are unlikely to be influenced by decision support when the effect of missing an abnormal case is sufficiently severe and driven by overriding protocols. Similarly, regulatory requirements around sepsis protocols are a major driver of repeated lactate testing that may not be amenable to decision support on predictable results. The results of this study can still inform the development of such regulatory requirements on the appropriate number and interval of screening tests that may otherwise be excessive or too rigid for individual cases. In predictable cases, the risk of false-positive test results (and adverse downstream effects) may be substantial.

These results can also provide foundational quantitative support for cost-effectiveness analysis. For example, if scaling the annual volume of predictable tests (predicted normal results) by their financial costs (eTable 10 in the Supplement), one could estimate annual savings by avoiding these tests. However, this saving should be carefully compared against potential harms and costs generated from missing the actually abnormal tests (false-negative results). In cases of panel test ordering, practitioners are often only interested in 1 or 2 components of panel tests at a time (eg, sodium level from a metabolic panel or hemoglobin level from a complete blood cell count). Most panel components may be predictably normal, but there could still be value in the overall order if there is sufficient uncertainty in at least 1 other clinically relevant component. Our separate predictions for each panel component in Figure 3 would allow practitioners to decide which components are relevant for their decision-making in future point-of-care information displays.

The results also allow us to systematically identify relevant factors that are predictive of each test result. This identification can inform simple rule-based clinical decision support based on factors including obvious elements, such as prior results, and less obvious ones, such as sex for ferritin status and surgical vs medical team for cerebrospinal fluid studies. eTables 11 through 16 in the Supplement include a full list of the most important features for predicting the normality of each laboratory test result.

### Limitations

Although we used conservative fixed-decision thresholds for clarity (targeting 95% NPV) in this proof-of-concept study, specific applications can undergo explicit decision analysis to assess the balance between risk and benefit. Even then, such future studies would require the foundation that we have established to assess the relative likelihood of different testing outcomes.

Assuming that the training data reflect the same distribution as the evaluation, intended application data distribution is an important limitation in any prediction model.^33^ Although we believe it may ultimately be more valuable to disseminate our underlying approach to undergo continuous learning and adaptation to local environments, we assessed model performance across multiple sites. Figure 4 shows that models trained at Stanford University can often still retain useful predictive performance when evaluated at UCSF and UMich, although these models will predictably underperform locally trained models. For example, the decrease in performance when predicting albumin levels at UMich with the model trained at Stanford University is likely associated with different underlying population distributions, including substantially different prevalences of normal albumin test results (16% at Stanford vs 57% at UMich). This finding is likely associated with different underlying population distributions, including substantially different prevalences of normal albumin test results (16% at Stanford vs 57% at UMich). On the other hand, the surprising increase of AUROC when applying the sodium model trained at Stanford University to UMich may indicate that sodium level was more excessively tested at UMich, making it easier to identify predictable repeated tests in their data.

Another factor that may lead to prediction failure is that the distribution of data could change over time. The point of refining decision support systems is to change ordering behavior, which is itself one of the most useful inputs into the predictive models. Consequently, we would recommend online learning algorithms^48^ that continuously adapt to practice changes rather than ever expecting to have a completed final model.

## Conclusions

The findings suggest that low-yield diagnostic testing is common and can be systematically identified through data-driven methods and patient context–aware predictions. Implementing continuous learning prediction models may help quantify the level of uncertainty and expected information gain from diagnostic tests explicitly, with potential to encourage useful testing and discourage low-value testing that can incur direct costs and indirect harms.

## Notes

Supplement. **eFigure 1.** Machine Learning Pipeline **eFigure 2.** ROC Curves for Stanford Standalone Labs **eFigure 3.** ROC Curves for Stanford Components **eFigure 4.** ROC Curves for UMich Standalone Labs **eFigure 5.** ROC Curves for UMich Components **eFigure 6.** ROC Curves for UCSF Standalone Labs **eFigure 7.** ROC Curves for UCSF Components **eTable 1.** Data Matrix Feature Summary **eTable 2.** Model Construction Summary **eTable 3.** Diagnostic Metrics for Top Stanford Standalone Labs **eTable 4.** Diagnostic Metrics for Common Stanford Components **eTable 5.** Diagnostic Metrics for Top UMich Standalone Labs **eTable 6.** Diagnostic Metrics for Common UMich Components **eTable 7.** Diagnostic Metrics for Top UCSF Standalone Labs **eTable 8.** Diagnostic Metrics for Common UCSF Components **eTable 9.** Diagnostic Metrics for Common Components in Transferability Study **eTable 10.** Medicare and Chargemaster Fees for Standalone Labs **eTable 11.** Top 3 Important Features for Top Stanford Standalone Labs **eTable 12.** Top 3 Important Features for Common Stanford Components **eTable 13.** Top 3 Important Features for Top UMich Standalone Labs **eTable 14.** Top 3 Important Features for Common UMich Components **eTable 15.** Top 3 Important Features for Top UCSF Standalone Labs **eTable 16.** Top 3 Important Features for Common UCSF Components **eMethods.** Technical Details of Machine Learning Algorithm
