# Errors in Figure 2, Figure 3, and Study Limitations

**DOI:** 10.1001/jamanetworkopen.2019.14190

**Published:** 2019-10-11

In the Original Investigation titled “Prevalence and Predictability of Low-Yield Inpatient Laboratory Diagnostic Tests,”^1^ published September 11, 2019, there was an error in the first row of Figure 2B. The label that appeared as “Peptide” should have been “C-peptide.” There was an error in Figure 3. The label for carbon dioxide was missing for the positives and negatives depicted. The sentence in the Limitations section, “This finding is likely associated with different underlying population distributions, including substantially different prevalences of normal albumin test results (16% at Stanford vs 57% at UMich),” was deleted as redundant. This article has been corrected.^1^
