# Supplementary material for: Prevalence and Predictability of Low-Yield Inpatient Laboratory Diagnostic Tests
Source: JAMA Netw Open. 2019 Sep 11;2(9):e1910967. doi: 10.1001/jamanetworkopen.2019.10967 (PMC6739729; doi:10.1001/jamanetworkopen.2019.10967)
Supplement: Supplement. — eFigure 1. Machine Learning Pipeline eFigure 2. ROC Curves for Stanford Standalone Labs eFigure 3. ROC Curves for Stanford Components eFigure 4. ROC Curves for UMich Standalone Labs eFigure 5. ROC Curves for UMich Components eFigure 6. ROC Curves for UCSF Standalone Labs eFigure 7. ROC Curves for UCSF Components eTable 1. Data Matrix Feature Summary eTable 2. Model Construction Summary eTable 3. Diagnostic Metrics for Top Stanford Standalone Labs eTable 4. Diagnostic Metrics for Common Stanford Components eTable 5. Diagnostic Metrics for Top UMich Standalone Labs eTable 6. Diagnostic Metrics for Common UMich Components eTable 7. Diagnostic Metrics for Top UCSF Standalone Labs eTable 8. Diagnostic Metrics for Common UCSF Components eTable 9. Diagnostic Metrics for Common Components in Transferability Study eTable 10. Medicare and Chargemaster Fees for Standalone Labs eTable 11. Top 3 Important Features for Top Stanford Standalone Labs eTable 12. Top 3 Important Features for Common Stanford Components eTable 13. Top 3 Important Features for Top UMich Standalone Labs eTable 14. Top 3 Important Features for Common UMich Components eTable 15. Top 3 Important Features for Top UCSF Standalone Labs eTable 16. Top 3 Important Features for Common UCSF Components eMethods. Technical Details of Machine Learning Algorithm [file jamanetwopen-2-e1910967-s001.pdf]

Supplementary Online Content

Xu S, Hom J, Balasubramanian S, et al. Prevalence and predictability of low-yield inpatient laboratory diagnostic tests. *JAMA Netw Open*. 2019;2(9):e1910967. doi:10.1001/jamanetworkopen.2019.10967

eFigure 1. Machine Learning Pipeline

eFigure 2. ROC Curves for Stanford Standalone Labs

eFigure 3. ROC Curves for Stanford Components

eFigure 4. ROC Curves for UMich Standalone Labs

eFigure 5. ROC Curves for UMich Components

eFigure 6. ROC Curves for UCSF Standalone Labs

eFigure 7. ROC Curves for UCSF Components

eTable 1. Data Matrix Feature Summary

eTable 2. Model Construction Summary

eTable 3. Diagnostic Metrics for Top Stanford Standalone Labs

eTable 4. Diagnostic Metrics for Common Stanford Components

eTable 5. Diagnostic Metrics for Top UMich Standalone Labs

eTable 6. Diagnostic Metrics for Common UMich Components

eTable 7. Diagnostic Metrics for Top UCSF Standalone Labs

eTable 8. Diagnostic Metrics for Common UCSF Components

eTable 9. Diagnostic Metrics for Common Components in Transferability Study

eTable 10. Medicare and Chargemaster Fees for Standalone Labs

eTable 11. Top 3 Important Features for Top Stanford Standalone Labs

eTable 12. Top 3 Important Features for Common Stanford Components

eTable 13. Top 3 Important Features for Top UMich Standalone Labs

eTable 14. Top 3 Important Features for Common UMich Components

eTable 15. Top 3 Important Features for Top UCSF Standalone Labs

eTable 16. Top 3 Important Features for Common UCSF Components

eMethods. Technical Details of Machine Learning Algorithm

This supplementary material has been provided by the authors to give readers additional information about their work.

© 2019 Xu S et al. *JAMA Network Open*.

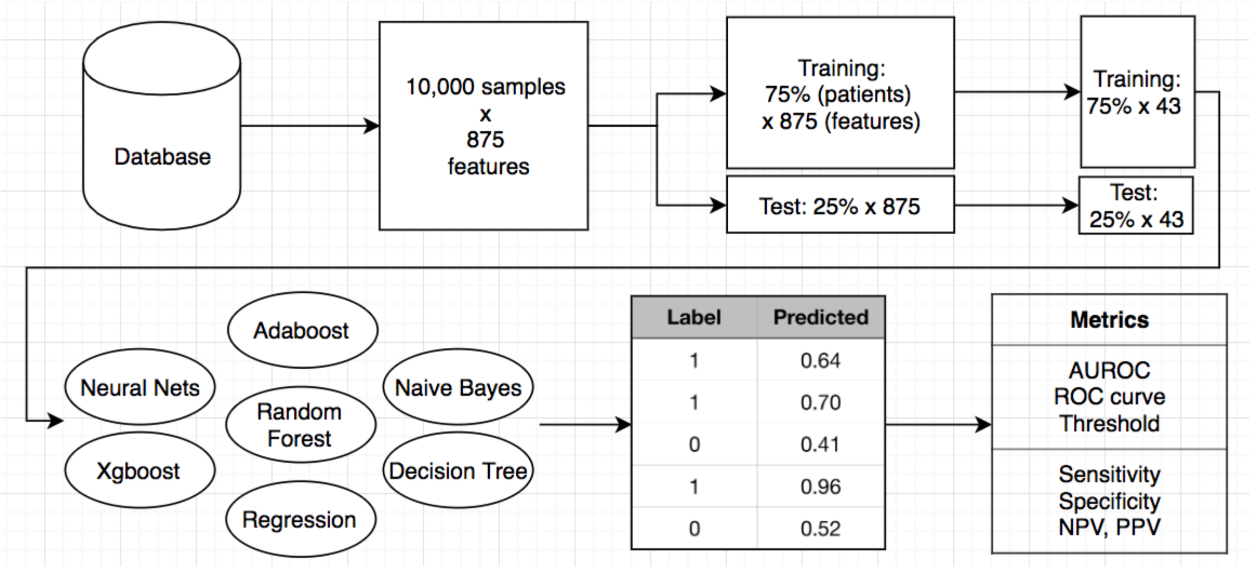

eFigure 1. Machine Learning Pipeline

Data processing, machine learning, and statistical analysis pipeline. When applied to a different dataset, an extra data extraction step will need to be implemented that extracts raw data (labs, diagnoses, demographics, encounters, patient info, treatment teams, etc.) from their database into Stanford-like columns (e.g. standardize 'DOB' into 'Birth'). The prediction output is a list of predicted normality scores, which were then compared against actual order results (labels). Label "0" denotes the laboratory test actually generates a normal/negative result while "1" means the result is abnormal/positive. ROC: receiver operating characteristic, AUROC (or C-statistic): Area Under the ROC curve, NPV: negative predictive value, PPV: positive predictive value.

© 2019 Xu S et al. *JAMA Network Open*.

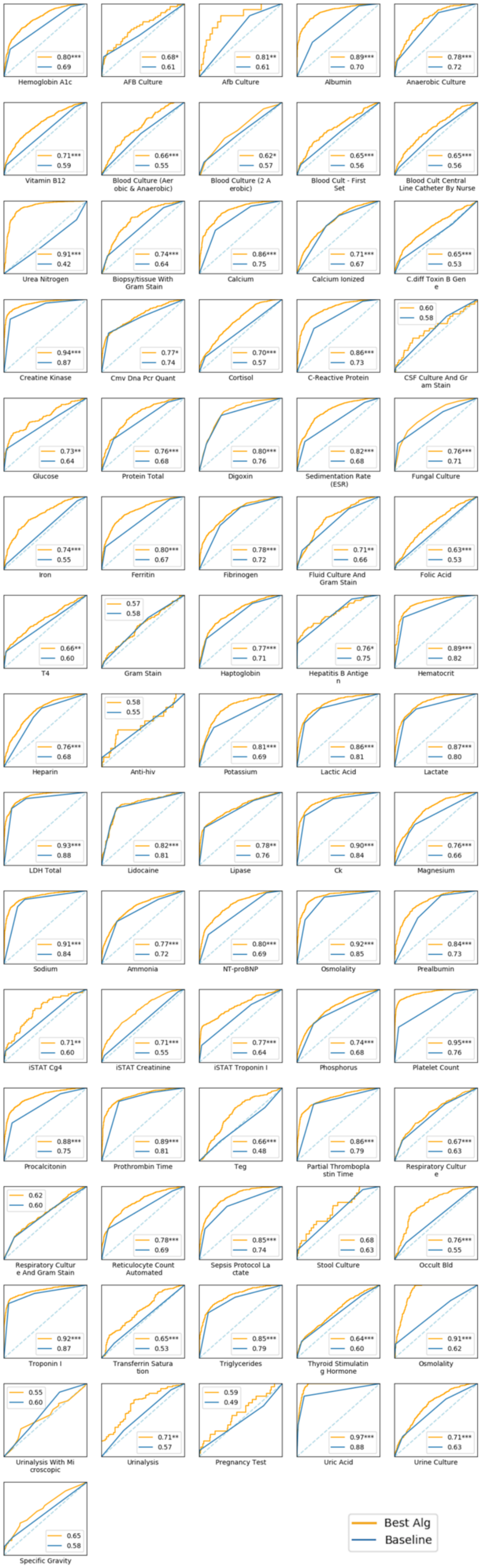

© 2019 Xu S et al. *JAMA Network Open*.

eFigure 2. ROC Curves for Stanford Standalone Labs

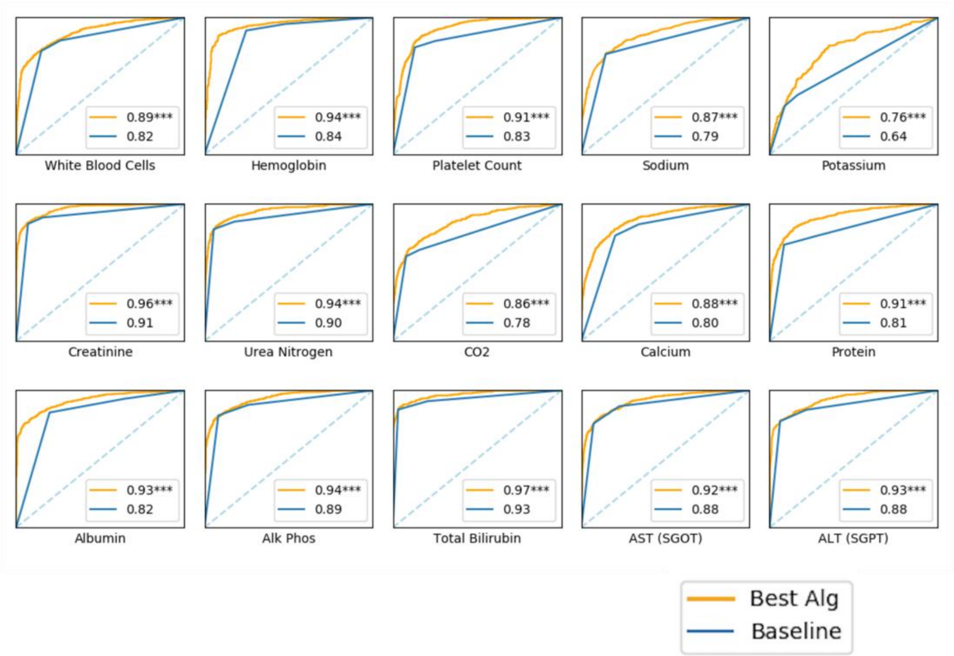

eFigure 3. ROC Curves for Stanford Components

© 2019 Xu S et al. *JAMA Network Open*.

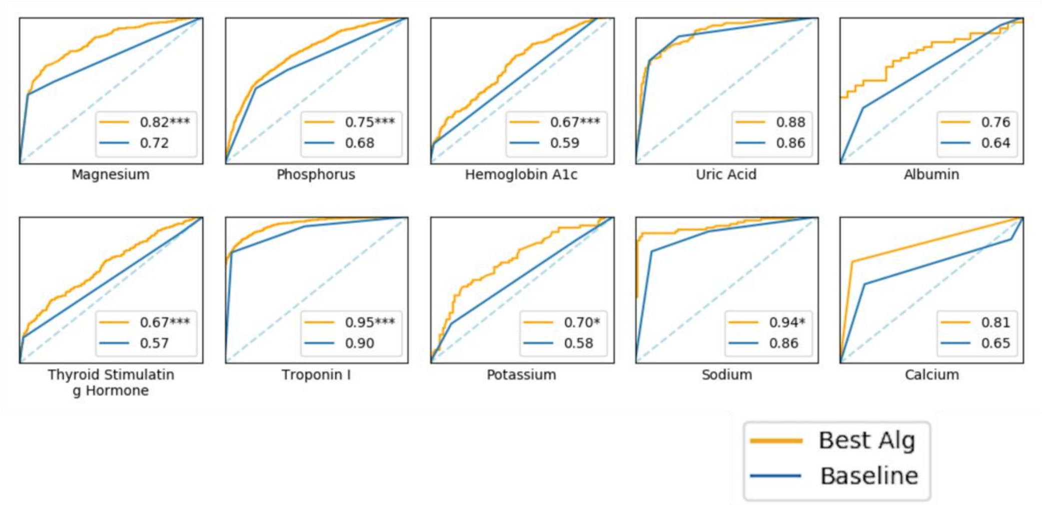

eFigure 4. ROC Curves for UMich Standalone Labs

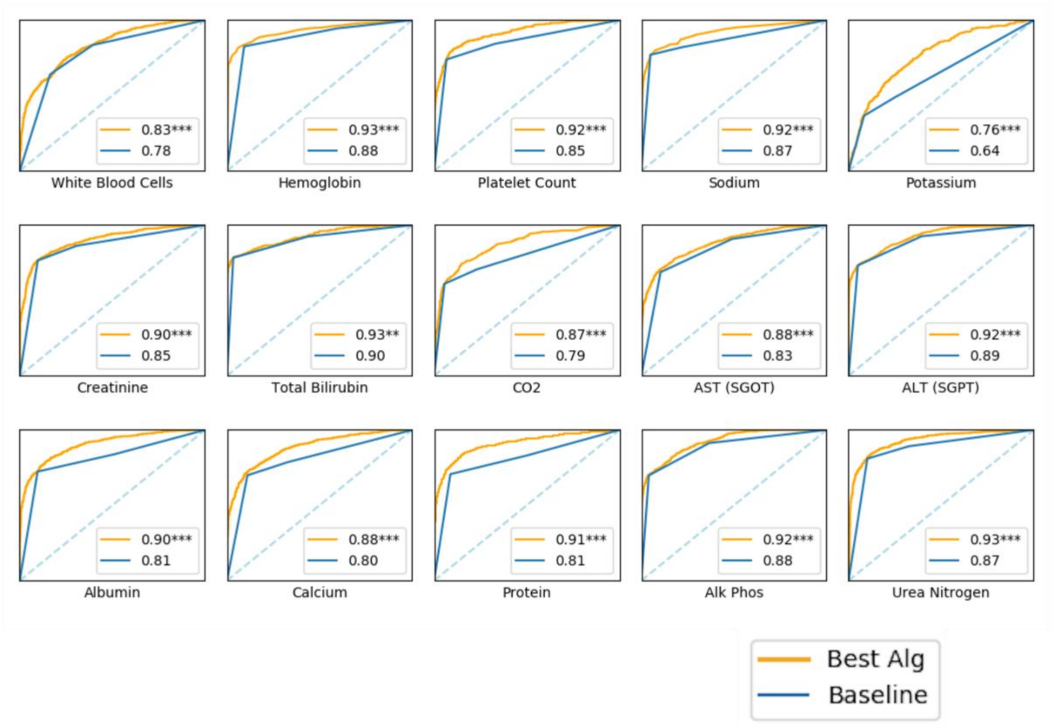

eFigure 5. ROC Curves for UMich Components

© 2019 Xu S et al. *JAMA Network Open*.

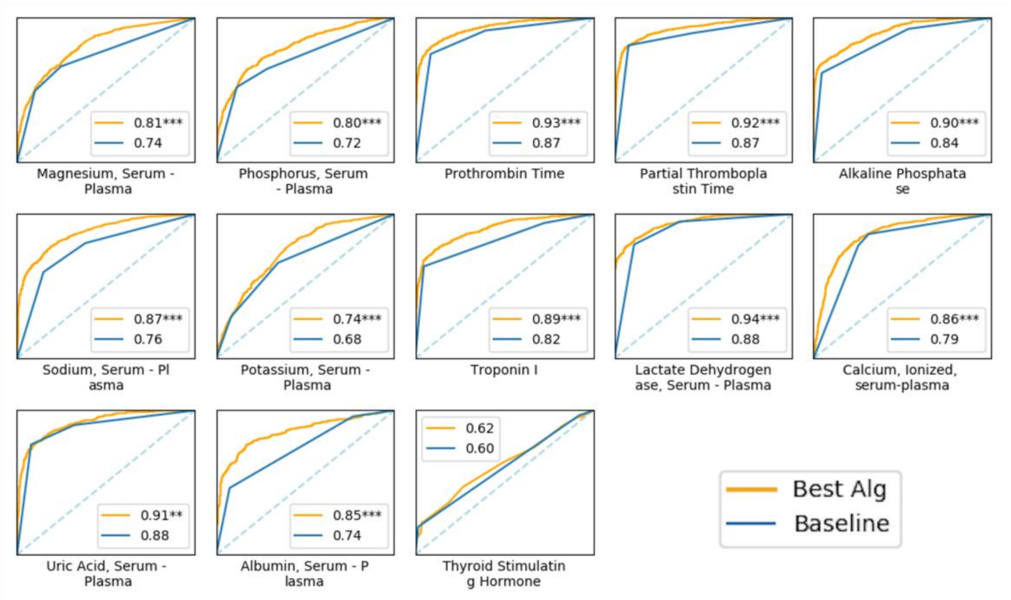

eFigure 6. ROC Curves for UCSF Standalone Labs

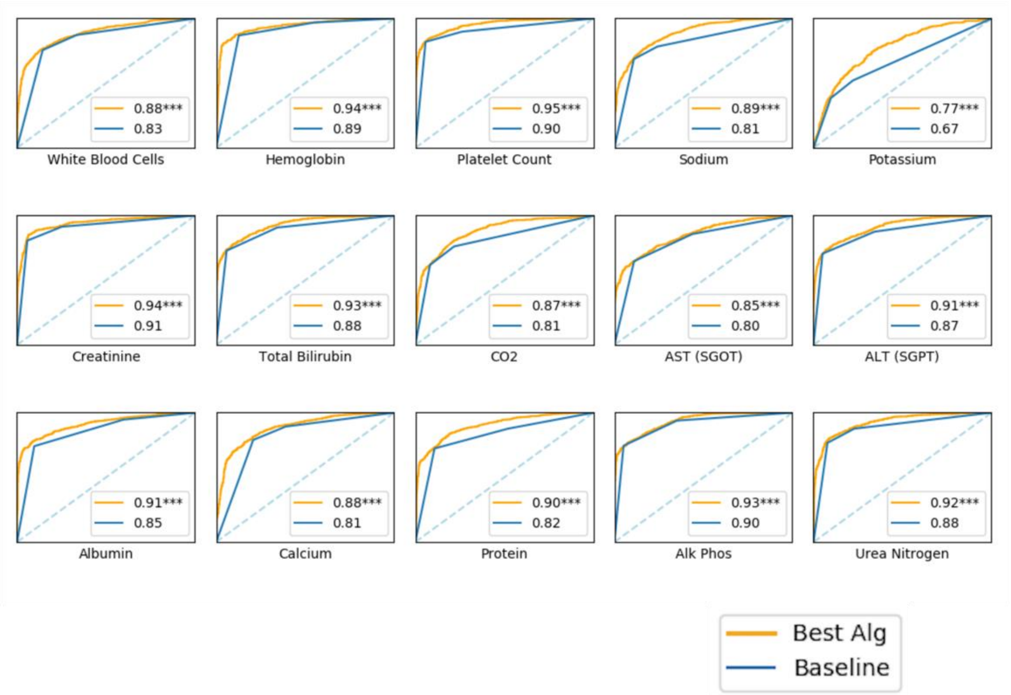

eFigure 7. ROC Curves for UCSF Components

© 2019 Xu S et al. *JAMA Network Open*.

category	type	features
Lab of Interest	binary	Normalilty of the Most Recent Order
	time	Time of Order (Month), Time of Order (Hour)<br>*Features modeled as nominal value, sine, and cosine.*
	time-binned counts	Order History<br>*Events represent prior orders of lab of interest.*
Demographics	integer	Age
	binary	Female, Male<br>Asian, Black, Hispanic/Latino, Native American, Pacific Islander, White Hispanic/Latino, White Non-Hispanic/Latino, Other Race, Unknown Race
Admission	integer	Days since Admission
Treatment Team	time-binned counts	Cardiology, Cardiovascular ICU, Coronary Care Unit, Hematology/Oncology, Medical ICU, Medicine, Neurology, Psychiatry, Surgery, Surgical ICU, Transplant, Trauma<br>*Events represent patient being treated by specialty.*
Flow Sheet	summary statistics	Diastolic Blood Pressure, FiO2, Glasgow Coma Scale Score, Pulse, Respiration, Systolic Blood Pressure, Temperature, Urine
Comorbidities	time-binned counts	Cerebrovascular Disease, Chronic Obstructive Pulmonary Disease (COPD), Congestive Heart Failure, Dementia, Diabetes, Diabetes Complications, Hemiplegia/Paraplegia, HIV/AIDS, Liver Damage (Mild), Liver Damage (Moderate to Severe), Malignancy, Metastatic Malignancy, Myocardial Infarction, Peptic Ulcer, Peripheral Vascular Disease, Renal Disease, Rheumatism<br>*Events represent comorbidity being added to problem list.*
Lab Results	summary statistics	Albumin, Arterial CO2 Partial Pressure, Arterial O2 Partial Pressure, Arterial pH, Blood Urea Nitrogen, C-reactive Protein, Calcium, CO2, Creatinine, Erythrocyte Sedimentation Rate, Hematocrit, Lactate, Platelet Count, Potassium, Sodium, Total Bilirubin, Troponin I, Venous CO2 Partial Pressure, Venous O2 Partial Pressure, Venous pH, White Blood Cell Count

eTable 1. Data Matrix Feature Summary

For each lab we studied, we constructed an M-by-N data matrix. Each of the N columns represents a single feature to potentially be used in the prediction of normal results for the lab of interest. In total, ~880 features were used for building each model (the precise value varied per lab test, based on the number of component results returned by the

© 2019 Xu S et al. *JAMA Network Open*.

clinical laboratory). The table shows the categories of features and their data types. Each of the time-binned counts represents an event aggregated at various time intervals (1, 2, 4, 7, 14, 30, 90, 180, 365, 730, and 1460 days before lab order) and days since last event. Each of the summary statistics represents a value aggregated over the past 3 (for vitals) or 14 days (for lab results) by count, normal count, minimum, maximum, median, standard deviation, first value, last value, slope, and days since first and last values.

Algorithm	Hyperparameters
AdaBoost	adaboost_algorithm: SAMME.R,<br>base_estimator: decision-tree,<br>class_weight: balanced,<br>learning_rate: [0.001, 0.01, 0.1, 1.0, 10.0],<br>n_estimators: [10, 20, 30, 40, 50]
Decision Tree	class_weight: balanced,<br>criterion: gini,<br>max_depth: [1, 2, 3, 4, 5, None],<br>max_features: [sqrt, log2, None],<br>max_leaf_nodes: None,<br>min_impurity_decrease: 0.0,<br>min_samples_leaf: [0.01, 0.1, 1.0, 10.0],<br>min_samples_split: [0.02, 0.2, 2, 20],<br>min_weight_fraction_leaf: 0.0,<br>presort: None,<br>splitter: best
Gaussian Naive Bayes	priors: [[0.0001, 0.9999], [0.001, 0.999], [0.01, 0.99], [0.05, 0.95], [0.1, 0.9], [0.25, 0.75], [0.5, 0.5], [0.75, 0.25], [0.9, 0.1], [0.95, 0.05], [0.99, 0.01], [0.999, 0.001], [0.9999, 0.0001]]
L1 Logistic Regression	C: [0.0001, 0.001, 0.01, 0.1, 1.0, 10.0, 100.0, 1000.0, 10000.0],<br>class_weight: balanced,<br>dual: False,<br>fit_intercept: True,<br>max_iter: 1024,<br>penalty: L1,<br>solver: SAGA,<br>tol: 0.0001
Neural Network	Activation: logistic, tanh, relu<br>Layer_sizes: (880, 10, 1), (880, 10, 10, 10, 1), (880, 10, 10, 10, 10, 10, 1)<br>Solver: lbgfs, sgd, adam
Random Forest	bootstrap: True,<br>n_estimators: [2, 5, 10, 15, 20, 25],<br>warm_start: False

© 2019 Xu S et al. *JAMA Network Open*.

	Other hyperparameters same as Decision Tree.
Regress and Round	M: [1, 2, 3, 4, 5]<br>Other hyperparameters same as L1 Logistic Regression.
XGBoost	Colsample_bytree: [0.6, 0.8, 1.0]<br>Learning_rate: [0.001, 0.01, 0.1, 1.0, 10.0]<br>Max_depth: [1, 2, 3, 4, 5]<br>Min_child_weight: [1, 5, 10]<br>Subsample: [0.6, 0.8, 1.0]

eTable 2. Model Construction Summary

Eight machine learning algorithms were applied to predict lab result. Each algorithm includes "hyperparameters" that can tune the performance of the algorithms. Hyperparameters with multiple values describe the hyperparameter search space, which was explored exhaustively and scored based on AUROC (C-statistics) through 10-fold cross validation.

Lab Test	Vol	AUROC	95% CI	Prev	Target NPV	NPV	PPV	Sens	Spec	TN	FN	TP	FP
Magnesium	4246	0.76	[0.74, 0.78]	26%	0.99	95%	29%	98%	17%	12%	0.60%	25%	62%
Magnesium	4246	0.76	[0.74, 0.78]	26%	0.95	91%	36%	86%	47%	35%	3.6%	22%	39%
Magnesium	4246	0.76	[0.74, 0.78]	26%	0.9	87%	43%	71%	67%	50%	7.6%	18%	24%
Magnesium	4246	0.76	[0.73, 0.78]	26%	0.8	79%	63%	29%	94%	70%	18%	7.5%	4.4%
Prothrombin Time	2244	0.89	[0.88, 0.91]	80%	0.99	92%	81%	100%	2.3%	0.45%	0.04%	80%	19%
Prothrombin Time	2244	0.89	[0.88, 0.91]	80%	0.95	85%	81%	100%	3.6%	0.70%	0.12%	80%	19%
Prothrombin Time	2244	0.89	[0.88, 0.91]	80%	0.9	82%	83%	99%	18%	3.4%	0.74%	80%	16%
Prothrombin Time	2244	0.89	[0.88, 0.90]	80%	0.8	69%	87%	96%	39%	7.6%	3.4%	77%	12%
Phosphorus	2120	0.74	[0.72, 0.76]	33%	0.9	84%	45%	78%	54%	36%	7.1%	26%	31%
Phosphorus	2120	0.74	[0.72, 0.76]	33%	0.95	88%	39%	91%	30%	20%	2.8%	30%	47%
Phosphorus	2120	0.74	[0.72, 0.76]	33%	0.8	77%	57%	52%	81%	55%	16%	17%	13%
Phosphorus	2120	0.74	[0.72, 0.76]	33%	0.99	88%	34%	98%	6.7%	4.5%	0.63%	32%	63%
Partial Thromboplastin Time	1471	0.86	[0.85, 0.87]	61%	0.99	90%	62%	100%	4.1%	1.6%	0.18%	61%	37%
Partial Thromboplastin Time	1471	0.86	[0.84, 0.87]	61%	0.9	81%	69%	96%	30%	12%	2.8%	59%	27%
Partial Thromboplastin Time	1471	0.86	[0.85, 0.87]	61%	0.8	74%	78%	87%	61%	23%	8.2%	53%	15%
Partial Thromboplastin Time	1471	0.86	[0.85, 0.87]	61%	0.95	87%	65%	98%	17%	6.5%	1.0%	60%	32%
Lactate	1230	0.87	[0.85, 0.88]	29%	0.8	77%	90%	28%	99%	71%	21%	8.0%	0.86%

© 2019 Xu S et al. *JAMA Network Open*.

Lactate	1230	0.87	[0.85, 0.88]	29%	0.95	91%	56%	82%	74%	53%	5.2%	23%	19%
Lactate	1230	0.87	[0.85, 0.89]	29%	0.99	97%	35%	98%	28%	20%	0.54%	28%	51%
Lactate	1230	0.87	[0.85, 0.89]	29%	0.9	87%	72%	65%	90%	64%	9.9%	19%	7.4%
Calcium Ionized	1197	0.72	[0.70, 0.74]	61%	0.95	90%	62%	100%	4.8%	1.9%	0.21%	61%	37%
Calcium Ionized	1197	0.72	[0.69, 0.74]	61%	0.9	82%	63%	99%	9.0%	3.5%	0.80%	60%	36%
Calcium Ionized	1197	0.72	[0.70, 0.74]	61%	0.8	71%	66%	93%	25%	10%	4.1%	57%	29%
Calcium Ionized	1197	0.72	[0.70, 0.74]	61%	0.99	96%	61%	100%	2.4%	0.93%	0.04%	61%	38%
Potassium	752	0.81	[0.79, 0.84]	12%	0.95	92%	40%	43%	91%	80%	7.0%	5.2%	7.9%
Potassium	752	0.81	[0.79, 0.84]	12%	0.8	88%	-	0	100%	88%	12%	0	0
Potassium	752	0.81	[0.79, 0.84]	12%	0.9	88%	64%	2.8%	100%	88%	12%	0.34%	0.19%
Potassium	752	0.81	[0.79, 0.84]	12%	0.99	97%	21%	89%	54%	48%	1.3%	11%	40%
Troponin I	534	0.92	[0.91, 0.93]	33%	0.99	95%	38%	98%	23%	16%	0.79%	32%	52%
Troponin I	534	0.92	[0.91, 0.93]	33%	0.95	93%	67%	88%	79%	53%	4.0%	29%	14%
Troponin I	534	0.92	[0.91, 0.93]	33%	0.9	89%	88%	76%	95%	64%	7.8%	25%	3.3%
Troponin I	534	0.92	[0.91, 0.93]	33%	0.8	79%	99%	45%	100%	67%	18%	15%	0.16%
LDH Total	455	0.93	[0.93, 0.94]	47%	0.8	79%	90%	72%	93%	50%	13%	34%	3.8%
LDH Total	455	0.93	[0.93, 0.94]	47%	0.9	90%	78%	90%	78%	42%	4.6%	42%	12%
LDH Total	455	0.93	[0.93, 0.94]	47%	0.99	98%	60%	99%	43%	23%	0.35%	46%	30%
LDH Total	455	0.93	[0.93, 0.94]	47%	0.95	95%	71%	96%	65%	35%	1.8%	45%	18%
Heparin	423	0.76	[0.74, 0.78]	63%	0.99	0	63%	100%	0	0	0.04%	63%	37%
Heparin	423	0.76	[0.74, 0.78]	63%	0.8	72%	71%	92%	37%	14%	5.2%	58%	24%
Heparin	423	0.76	[0.74, 0.78]	63%	0.9	74%	68%	95%	24%	8.9%	3.2%	60%	28%
Heparin	423	0.76	[0.74, 0.78]	63%	0.95	0	63%	100%	0	0	0.04%	63%	37%
Urinalysis	417	0.71	[0.63, 0.80]	80%	0.95	-	80%	100%	0	0	0	80%	20%
Urinalysis	417	0.71	[0.63, 0.79]	80%	0.8	-	80%	100%	0	0	0	80%	20%
Urinalysis	417	0.71	[0.63, 0.79]	80%	0.99	-	80%	100%	0	0	0	80%	20%
Urinalysis	417	0.71	[0.63, 0.79]	80%	0.9	-	80%	100%	0	0	0	80%	20%
Blood Culture (Aerobic & Anaerobic)	400	0.66	[0.61, 0.71]	8.1%	0.8	92%	-	0	100%	92%	8.1%	0	0
Blood Culture (Aerobic & Anaerobic)	400	0.66	[0.61, 0.71]	8.1%	0.9	92%	-	0	100%	92%	8.1%	0	0
Blood Culture (Aerobic & Anaerobic)	400	0.66	[0.61, 0.71]	8.1%	0.95	93%	16%	16%	93%	85%	6.8%	1.3%	6.6%
Blood Culture (Aerobic & Anaerobic)	400	0.66	[0.61, 0.71]	8.1%	0.99	94%	14%	45%	76%	70%	4.5%	3.6%	22%
Blood Culture (2 Aerobic)	371	0.62	[0.58, 0.67]	9.1%	0.95	93%	12%	61%	54%	49%	3.6%	5.6%	42%
Blood Culture (2 Aerobic)	371	0.62	[0.58, 0.67]	9.1%	0.8	91%	-	0	100%	91%	9.1%	0	0
Blood Culture (2 Aerobic)	371	0.62	[0.58, 0.67]	9.1%	0.9	91%	-	0	100%	91%	9.1%	0	0

© 2019 Xu S et al. *JAMA Network Open*.

Blood Culture (2 Aerobic)	371	0.62	[0.57, 0.67]	9.1%	0.99	-	9.1%	100%	0	0	0	9.1%	91%
Sodium	361	0.92	[0.91, 0.93]	57%	0.99	100%	58%	100%	3.1%	1.4%	0	57%	42%
Sodium	361	0.92	[0.91, 0.93]	57%	0.95	93%	66%	98%	35%	15%	1.1%	56%	28%
Sodium	361	0.92	[0.91, 0.93]	57%	0.8	76%	91%	78%	90%	39%	12%	45%	4.5%
Sodium	361	0.92	[0.91, 0.93]	57%	0.9	87%	79%	92%	68%	29%	4.5%	52%	14%
Lidocaine	315	0.83	[0.79, 0.86]	23%	0.99	94%	39%	89%	57%	44%	2.6%	21%	33%
Lidocaine	315	0.83	[0.79, 0.86]	23%	0.95	91%	55%	73%	82%	63%	6.3%	17%	14%
Lidocaine	315	0.83	[0.79, 0.86]	23%	0.8	77%	100%	0.62%	100%	77%	23%	0.14%	0
Lidocaine	315	0.83	[0.79, 0.86]	23%	0.9	83%	61%	35%	93%	71%	15%	8.2%	5.3%
Hematocrit	288	0.9	[0.88, 0.92]	93%	0.99	100%	93%	100%	1.0%	0.08%	0	93%	7.2%
Hematocrit	288	0.9	[0.87, 0.92]	93%	0.8	50%	94%	99%	16%	1.1%	1.1%	92%	6.1%
Hematocrit	288	0.9	[0.88, 0.92]	93%	0.9	44%	93%	99%	7.9%	0.57%	0.72%	92%	6.7%
Hematocrit	288	0.9	[0.88, 0.92]	93%	0.95	58%	93%	100%	3.7%	0.27%	0.19%	93%	7.0%
Urine Culture	257	0.71	[0.68, 0.74]	36%	0.99	100%	36%	100%	2.2%	1.4%	0	36%	63%
Urine Culture	257	0.71	[0.68, 0.74]	36%	0.95	90%	41%	95%	24%	15%	1.7%	34%	49%
Urine Culture	257	0.71	[0.68, 0.74]	36%	0.9	84%	44%	86%	40%	26%	5.0%	31%	39%
Urine Culture	257	0.71	[0.68, 0.74]	36%	0.8	75%	52%	58%	71%	45%	15%	21%	19%
Urinalysis With Microscopic	246	0.63	[0.57, 0.70]	72%	0.9	40%	75%	86%	24%	6.6%	10%	62%	21%
Urinalysis With Microscopic	246	0.63	[0.56, 0.70]	72%	0.8	35%	75%	78%	32%	8.8%	16%	56%	19%
Urinalysis With Microscopic	246	0.63	[0.57, 0.70]	72%	0.95	48%	75%	93%	17%	4.7%	5.0%	67%	23%
Urinalysis With Microscopic	246	0.63	[0.57, 0.70]	72%	0.99	43%	73%	97%	6.8%	1.9%	2.5%	70%	26%
Uric Acid	229	0.97	[0.96, 0.98]	3.8%	0.99	98%	51%	50%	98%	94%	1.9%	1.9%	1.8%
Uric Acid	229	0.97	[0.96, 0.98]	3.8%	0.95	96%	-	0	100%	96%	3.8%	0	0
Uric Acid	229	0.97	[0.96, 0.98]	3.8%	0.8	96%	-	0	100%	96%	3.8%	0	0
Uric Acid	229	0.97	[0.96, 0.98]	3.8%	0.9	96%	-	0	100%	96%	3.8%	0	0
Hemoglobin A1c	225	0.81	[0.79, 0.82]	59%	0.95	87%	63%	99%	14%	5.8%	0.86%	58%	35%
Hemoglobin A1c	225	0.81	[0.79, 0.82]	59%	0.99	93%	61%	100%	6.8%	2.8%	0.20%	59%	38%
Hemoglobin A1c	225	0.81	[0.79, 0.82]	59%	0.9	85%	66%	97%	28%	11%	2.0%	57%	29%
Hemoglobin A1c	225	0.81	[0.79, 0.82]	59%	0.8	73%	74%	86%	55%	23%	8.4%	51%	18%
Sepsis Protocol Lactate	187	0.86	[0.84, 0.88]	17%	0.99	97%	28%	92%	52%	44%	1.3%	15%	40%
Sepsis Protocol Lactate	187	0.86	[0.84, 0.88]	17%	0.8	83%	-	0	100%	83%	17%	0	0
Sepsis Protocol Lactate	187	0.86	[0.84, 0.88]	17%	0.9	87%	80%	30%	98%	82%	12%	5.0%	1.3%
Sepsis Protocol Lactate	187	0.86	[0.84, 0.88]	17%	0.95	92%	55%	60%	90%	75%	6.7%	10%	8.4%
iSTAT Troponin I	184	0.79	[0.75, 0.82]	13%	0.99	95%	16%	91%	26%	23%	1.1%	12%	64%
iSTAT Troponin I	184	0.79	[0.75, 0.82]	13%	0.95	93%	55%	54%	93%	81%	6.2%	7.1%	5.9%
iSTAT Troponin I	184	0.79	[0.75, 0.82]	13%	0.9	87%	93%	4.6%	100%	87%	13%	0.62%	0.05%

© 2019 Xu S et al. *JAMA Network Open*.

iSTAT Troponin I	184	0.79	[0.75, 0.82]	13%	0.8	87%	-	0	100%	87%	13%	0	0
Platelet Count	174	0.95	[0.94, 0.96]	44%	0.99	98%	57%	99%	41%	23%	0.44%	43%	33%
Platelet Count	174	0.95	[0.94, 0.96]	44%	0.95	92%	76%	92%	78%	44%	3.6%	40%	12%
Platelet Count	174	0.95	[0.94, 0.96]	44%	0.9	89%	90%	85%	92%	52%	6.6%	37%	4.2%
Platelet Count	174	0.95	[0.94, 0.96]	44%	0.8	80%	98%	68%	99%	55%	14%	30%	0.67%
Lipase	174	0.79	[0.77, 0.82]	23%	0.99	93%	26%	96%	17%	13%	0.98%	22%	64%
Lipase	174	0.79	[0.77, 0.81]	23%	0.95	91%	35%	82%	53%	41%	4.3%	19%	36%
Lipase	174	0.79	[0.77, 0.82]	23%	0.8	80%	92%	15%	100%	76%	20%	3.6%	0.30%
Lipase	174	0.79	[0.77, 0.82]	23%	0.9	88%	55%	61%	85%	65%	9.1%	14%	11%
Procalcitonin	174	0.89	[0.88, 0.90]	52%	0.95	91%	61%	97%	34%	17%	1.6%	50%	32%
Procalcitonin	174	0.89	[0.88, 0.90]	52%	0.9	85%	73%	89%	65%	31%	5.5%	46%	17%
Procalcitonin	174	0.89	[0.88, 0.90]	52%	0.8	77%	86%	75%	87%	42%	13%	39%	6.2%
Procalcitonin	174	0.89	[0.88, 0.90]	52%	0.99	96%	53%	100%	5.7%	2.8%	0.12%	52%	46%
Lactic Acid	152	0.87	[0.85, 0.88]	25%	0.9	87%	72%	59%	92%	69%	10%	15%	5.8%
Lactic Acid	152	0.87	[0.85, 0.88]	25%	0.8	79%	95%	20%	100%	75%	20%	5.0%	0.25%
Lactic Acid	152	0.87	[0.85, 0.88]	25%	0.95	92%	54%	79%	78%	58%	5.3%	20%	17%
Lactic Acid	152	0.87	[0.85, 0.88]	25%	0.99	97%	34%	97%	36%	27%	0.85%	24%	48%
Fibrinogen	148	0.78	[0.76, 0.80]	61%	0.99	100%	61%	100%	0.41%	0.16%	0	61%	39%
Fibrinogen	148	0.78	[0.76, 0.80]	61%	0.95	79%	63%	98%	11%	4.1%	1.1%	60%	35%
Fibrinogen	148	0.78	[0.77, 0.80]	61%	0.9	76%	67%	95%	27%	10%	3.3%	58%	29%
Fibrinogen	148	0.78	[0.76, 0.80]	61%	0.8	74%	72%	90%	44%	17%	6.1%	55%	22%
Thyroid Stimulating Hormone	145	0.64	[0.62, 0.67]	27%	0.9	81%	34%	67%	52%	38%	9.0%	18%	35%
Thyroid Stimulating Hormone	145	0.64	[0.62, 0.67]	27%	0.8	77%	54%	26%	92%	67%	20%	7.1%	6.0%
Thyroid Stimulating Hormone	145	0.64	[0.62, 0.67]	27%	0.99	84%	27%	100%	0.87%	0.63%	0.12%	27%	72%
Thyroid Stimulating Hormone	145	0.64	[0.62, 0.67]	27%	0.95	83%	30%	87%	24%	18%	3.7%	24%	55%
Creatine Kinase	126	0.94	[0.93, 0.95]	48%	0.9	87%	88%	86%	89%	47%	6.8%	41%	5.6%
Creatine Kinase	126	0.94	[0.93, 0.95]	48%	0.8	80%	96%	73%	97%	51%	13%	35%	1.4%
Creatine Kinase	126	0.94	[0.93, 0.95]	48%	0.99	95%	66%	97%	55%	29%	1.5%	46%	24%
Creatine Kinase	126	0.94	[0.93, 0.95]	48%	0.95	91%	79%	92%	78%	41%	4.0%	44%	12%
C-Reactive Protein	125	0.86	[0.84, 0.88]	87%	0.99	82%	87%	100%	3.9%	0.52%	0.11%	87%	13%
C-Reactive Protein	125	0.86	[0.84, 0.88]	87%	0.8	68%	89%	98%	22%	3.0%	1.4%	85%	10%
C-Reactive Protein	125	0.86	[0.84, 0.88]	87%	0.95	81%	87%	100%	6.1%	0.81%	0.18%	86%	13%
C-Reactive Protein	125	0.86	[0.84, 0.88]	87%	0.9	72%	88%	99%	12%	1.6%	0.59%	86%	12%
NT-proBNP	122	0.81	[0.79, 0.83]	82%	0.8	74%	84%	99%	17%	3.2%	1.1%	81%	15%
NT-proBNP	122	0.81	[0.79, 0.83]	82%	0.9	76%	83%	99%	9.4%	1.7%	0.53%	81%	17%
NT-proBNP	122	0.81	[0.79, 0.83]	82%	0.95	70%	82%	100%	1.6%	0.29%	0.12%	82%	18%
NT-proBNP	122	0.81	[0.79, 0.83]	82%	0.99	71%	82%	100%	1.1%	0.20%	0.08%	82%	18%

© 2019 Xu S et al. *JAMA Network Open*.

Triglycerides	105	0.86	[0.84, 0.87]	42%	0.8	76%	84%	61%	91%	53%	17%	26%	5.0%
Triglycerides	105	0.86	[0.84, 0.87]	42%	0.9	84%	66%	82%	69%	40%	7.5%	35%	18%
Triglycerides	105	0.86	[0.84, 0.87]	42%	0.95	92%	54%	95%	41%	24%	2.1%	40%	34%
Triglycerides	105	0.86	[0.84, 0.87]	42%	0.99	95%	47%	99%	19%	11%	0.55%	42%	46%
CK, MB	96	0.9	[0.88, 0.91]	49%	0.8	79%	86%	75%	88%	45%	12%	37%	5.9%
CK, MB	96	0.9	[0.88, 0.91]	49%	0.9	86%	74%	88%	70%	36%	5.7%	43%	15%
CK, MB	96	0.9	[0.88, 0.91]	49%	0.95	91%	64%	95%	48%	24%	2.4%	46%	27%
CK, MB	96	0.9	[0.88, 0.91]	49%	0.99	96%	53%	99%	15%	7.7%	0.35%	49%	43%
C.diff Toxin B Gene	95	0.65	[0.62, 0.68]	18%	0.8	82%	-	0	100%	82%	18%	0	0
C.diff Toxin B Gene	95	0.65	[0.62, 0.68]	18%	0.9	87%	27%	55%	67%	55%	8.2%	9.9%	27%
C.diff Toxin B Gene	95	0.65	[0.62, 0.68]	18%	0.95	89%	21%	85%	28%	23%	2.7%	15%	59%
C.diff Toxin B Gene	95	0.65	[0.62, 0.68]	18%	0.99	80%	18%	99%	1.2%	0.96%	0.24%	18%	81%
Osmolality	88	0.92	[0.91, 0.93]	53%	0.8	76%	91%	75%	91%	43%	13%	40%	4.0%
Osmolality	88	0.92	[0.91, 0.93]	53%	0.9	88%	82%	91%	77%	36%	4.9%	48%	11%
Osmolality	88	0.92	[0.91, 0.93]	53%	0.95	94%	70%	97%	53%	25%	1.7%	52%	22%
Osmolality	88	0.92	[0.91, 0.93]	53%	0.99	96%	56%	100%	11%	5.4%	0.22%	53%	41%
Respiratory Culture And Gram Stain	87	0.62	[0.58, 0.65]	35%	0.99	80%	35%	100%	0.59%	0.38%	0.10%	34%	65%
Respiratory Culture And Gram Stain	87	0.62	[0.58, 0.65]	35%	0.95	92%	35%	100%	1.8%	1.2%	0.10%	34%	64%
Respiratory Culture And Gram Stain	87	0.62	[0.58, 0.65]	35%	0.9	78%	36%	92%	15%	9.5%	2.7%	32%	56%
Respiratory Culture And Gram Stain	87	0.62	[0.58, 0.65]	35%	0.8	72%	42%	55%	61%	40%	15%	19%	26%
Sedimentation Rate (ESR)	85	0.83	[0.81, 0.85]	69%	0.99	100%	70%	100%	2.0%	0.62%	0	69%	30%
Sedimentation Rate (ESR)	85	0.83	[0.81, 0.84]	69%	0.95	86%	73%	99%	19%	5.9%	1.0%	68%	25%
Sedimentation Rate (ESR)	85	0.83	[0.81, 0.84]	69%	0.9	80%	75%	97%	30%	9.1%	2.3%	67%	22%
Sedimentation Rate (ESR)	85	0.83	[0.81, 0.85]	69%	0.8	70%	78%	92%	41%	13%	5.4%	64%	18%
Ferritin	82	0.81	[0.79, 0.82]	62%	0.95	91%	63%	100%	4.4%	1.7%	0.16%	62%	36%
Ferritin	82	0.81	[0.79, 0.82]	62%	0.8	73%	72%	91%	42%	16%	5.8%	57%	22%
Ferritin	82	0.81	[0.79, 0.82]	62%	0.9	80%	66%	97%	17%	6.6%	1.6%	61%	31%
Ferritin	82	0.81	[0.79, 0.82]	62%	0.99	93%	63%	100%	2.6%	0.98%	0.08%	62%	37%
Albumin	78	0.9	[0.88, 0.91]	89%	0.95	75%	90%	99%	15%	1.7%	0.57%	88%	9.7%
Albumin	78	0.9	[0.88, 0.91]	89%	0.9	71%	91%	99%	26%	2.9%	1.2%	87%	8.5%
Albumin	78	0.9	[0.88, 0.91]	89%	0.8	60%	92%	97%	35%	4.0%	2.6%	86%	7.4%
Albumin	78	0.9	[0.87, 0.91]	89%	0.99	74%	89%	100%	5.0%	0.57%	0.20%	88%	11%
Ammonia	77	0.78	[0.76, 0.79]	59%	0.8	73%	69%	90%	39%	16%	5.9%	54%	25%
Ammonia	77	0.78	[0.76, 0.79]	59%	0.9	78%	63%	97%	17%	6.7%	1.9%	58%	34%

© 2019 Xu S et al. *JAMA Network Open*.

Ammonia	77	0.78	[0.76, 0.80]	59%	0.95	80%	61%	99%	8.3%	3.4%	0.85%	59%	37%
Ammonia	77	0.78	[0.76, 0.79]	59%	0.99	77%	60%	99%	3.4%	1.4%	0.42%	59%	39%
Specific Gravity	76	0.67	[0.54, 0.79]	0.85%	0.99	99%	-	0	100%	99%	0.85%	0	0
Specific Gravity	76	0.67	[0.55, 0.79]	0.85%	0.95	99%	-	0	100%	99%	0.85%	0	0
Specific Gravity	76	0.67	[0.55, 0.78]	0.85%	0.9	99%	-	0	100%	99%	0.85%	0	0
Specific Gravity	76	0.67	[0.54, 0.79]	0.85%	0.8	99%	-	0	100%	99%	0.85%	0	0
Fungal Culture	74	0.76	[0.73, 0.79]	18%	0.8	82%	-	0	100%	82%	18%	0	0
Fungal Culture	74	0.76	[0.73, 0.80]	18%	0.9	93%	28%	81%	54%	45%	3.5%	15%	37%
Fungal Culture	74	0.76	[0.72, 0.80]	18%	0.95	-	18%	100%	0	0	0	18%	82%
Fungal Culture	74	0.76	[0.73, 0.80]	18%	0.99	-	18%	100%	0	0	0	18%	82%
Haptoglobin	71	0.77	[0.75, 0.79]	43%	0.8	74%	65%	66%	73%	42%	14%	28%	15%
Haptoglobin	71	0.77	[0.75, 0.79]	43%	0.9	83%	53%	89%	40%	23%	4.8%	38%	34%
Haptoglobin	71	0.77	[0.75, 0.79]	43%	0.95	88%	47%	96%	20%	11%	1.6%	41%	46%
Haptoglobin	71	0.77	[0.75, 0.79]	43%	0.99	97%	44%	100%	5.5%	3.2%	0.09%	43%	54%
Anaerobic Culture	69	0.78	[0.75, 0.82]	13%	0.9	90%	51%	25%	97%	84%	9.5%	3.2%	3.0%
Anaerobic Culture	69	0.78	[0.75, 0.82]	13%	0.99	99%	17%	97%	33%	29%	0.41%	12%	58%
Anaerobic Culture	69	0.78	[0.75, 0.82]	13%	0.95	94%	28%	64%	76%	66%	4.6%	8.1%	21%
Anaerobic Culture	69	0.78	[0.75, 0.82]	13%	0.8	87%	-	0	100%	87%	13%	0	0
Fluid Culture And Gram Stain	69	0.73	[0.69, 0.76]	26%	0.99	91%	29%	94%	20%	15%	1.5%	25%	59%
Fluid Culture And Gram Stain	69	0.73	[0.69, 0.76]	26%	0.95	85%	41%	67%	66%	49%	8.7%	17%	25%
Fluid Culture And Gram Stain	69	0.73	[0.69, 0.76]	26%	0.9	80%	50%	42%	85%	63%	15%	11%	11%
Fluid Culture And Gram Stain	69	0.73	[0.69, 0.77]	26%	0.8	76%	67%	10%	98%	73%	23%	2.7%	1.4%
Cmv Dna Pcr Quant	66	0.79	[0.76, 0.82]	27%	0.8	79%	90%	28%	99%	72%	20%	7.7%	0.85%
Cmv Dna Pcr Quant	66	0.79	[0.76, 0.82]	27%	0.9	85%	61%	58%	86%	63%	11%	16%	10%
Cmv Dna Pcr Quant	66	0.79	[0.76, 0.82]	27%	0.95	88%	44%	76%	63%	46%	6.5%	21%	27%
Cmv Dna Pcr Quant	66	0.79	[0.76, 0.82]	27%	0.99	92%	31%	95%	21%	15%	1.3%	26%	57%
Blood Cult Central Line Catheter By Nurse	62	0.65	[0.60, 0.69]	11%	0.95	90%	20%	19%	90%	80%	8.9%	2.1%	8.5%
Blood Cult Central Line Catheter By Nurse	62	0.65	[0.60, 0.70]	11%	0.8	89%	-	0	100%	89%	11%	0	0
Blood Cult Central Line Catheter By Nurse	62	0.65	[0.60, 0.70]	11%	0.9	89%	100%	2.1%	100%	89%	11%	0.23%	0
Blood Cult Central Line Catheter By Nurse	62	0.65	[0.61, 0.69]	11%	0.99	93%	15%	68%	52%	46%	3.6%	7.4%	43%
T4, FREE	61	0.68	[0.64, 0.71]	11%	0.9	89%	100%	0.34%	100%	89%	11%	0.04%	0
T4, FREE	61	0.68	[0.64, 0.71]	11%	0.95	92%	28%	37%	88%	78%	7.1%	4.2%	11%
T4, FREE	61	0.68	[0.64, 0.71]	11%	0.99	95%	13%	90%	24%	21%	1.1%	10%	68%
T4, FREE	61	0.68	[0.64, 0.71]	11%	0.8	89%	-	0	100%	89%	11%	0	0

© 2019 Xu S et al. *JAMA Network Open*.

Transferrin Saturation	60	0.65	[0.60, 0.70]	88%	0.8	-	88%	100%	0	0	0	88%	12%
Transferrin Saturation	60	0.65	[0.60, 0.70]	88%	0.99	-	88%	100%	0	0	0	88%	12%
Transferrin Saturation	60	0.65	[0.60, 0.70]	88%	0.9	-	88%	100%	0	0	0	88%	12%
Transferrin Saturation	60	0.65	[0.60, 0.70]	88%	0.95	-	88%	100%	0	0	0	88%	12%
Vitamin B12	60	0.72	[0.70, 0.74]	32%	0.8	77%	54%	50%	80%	54%	16%	16%	14%
Vitamin B12	60	0.72	[0.70, 0.74]	32%	0.9	85%	40%	86%	39%	26%	4.5%	28%	42%
Vitamin B12	60	0.72	[0.70, 0.74]	32%	0.95	87%	35%	96%	13%	8.8%	1.3%	31%	59%
Vitamin B12	60	0.72	[0.70, 0.74]	32%	0.99	93%	32%	100%	0.82%	0.56%	0.04%	32%	67%
Blood Cult - First Set	60	0.67	[0.62, 0.71]	11%	0.99	92%	18%	54%	70%	62%	5.0%	6.0%	27%
Blood Cult - First Set	60	0.67	[0.62, 0.71]	11%	0.9	89%	0	0	100%	89%	11%	0	0.08%
Blood Cult - First Set	60	0.67	[0.62, 0.72]	11%	0.8	89%	-	0	100%	89%	11%	0	0
Blood Cult - First Set	60	0.67	[0.62, 0.71]	11%	0.95	90%	25%	16%	94%	84%	9.3%	1.8%	5.2%
Prealbumin	59	0.85	[0.83, 0.86]	77%	0.99	77%	77%	100%	1.9%	0.45%	0.14%	77%	23%
Prealbumin	59	0.85	[0.83, 0.86]	77%	0.95	90%	79%	100%	13%	3.0%	0.32%	76%	20%
Prealbumin	59	0.85	[0.83, 0.86]	77%	0.9	85%	81%	99%	23%	5.4%	0.95%	76%	18%
Prealbumin	59	0.85	[0.83, 0.86]	77%	0.8	79%	84%	97%	39%	9.0%	2.4%	74%	14%
Osmolality, Urine	58	0.92	[0.90, 0.93]	7.9%	0.95	94%	50%	22%	98%	90%	6.1%	1.8%	1.8%
Osmolality, Urine	58	0.92	[0.90, 0.93]	7.9%	0.99	97%	34%	70%	88%	81%	2.4%	5.5%	11%
Osmolality, Urine	58	0.92	[0.90, 0.93]	7.9%	0.9	92%	-	0	100%	92%	7.9%	0	0
Osmolality, Urine	58	0.92	[0.90, 0.93]	7.9%	0.8	92%	-	0	100%	92%	7.9%	0	0
Digoxin	55	0.8	[0.77, 0.83]	22%	0.99	-	22%	100%	0	0	0	22%	78%
Digoxin	55	0.8	[0.77, 0.83]	22%	0.95	95%	36%	89%	56%	44%	2.4%	19%	35%
Digoxin	55	0.8	[0.77, 0.83]	22%	0.9	92%	44%	78%	72%	57%	4.7%	17%	22%
Digoxin	55	0.8	[0.77, 0.83]	22%	0.8	81%	56%	19%	96%	75%	17%	4.1%	3.2%
Reticulocyte Count Automated	50	0.79	[0.77, 0.81]	63%	0.9	81%	70%	96%	28%	10%	2.5%	61%	27%
Reticulocyte Count Automated	50	0.79	[0.77, 0.81]	63%	0.99	81%	64%	100%	3.3%	1.2%	0.29%	63%	36%
Reticulocyte Count Automated	50	0.79	[0.77, 0.81]	63%	0.8	71%	73%	89%	45%	16%	6.9%	56%	20%
Reticulocyte Count Automated	50	0.79	[0.77, 0.81]	63%	0.95	85%	66%	99%	11%	4.2%	0.74%	62%	33%
Cortisol	48	0.71	[0.68, 0.73]	24%	0.8	77%	78%	6.5%	99%	75%	23%	1.6%	0.45%
Cortisol	48	0.71	[0.69, 0.73]	24%	0.9	83%	38%	52%	73%	56%	12%	12%	20%
Cortisol	48	0.71	[0.69, 0.73]	24%	0.95	88%	33%	78%	50%	38%	5.3%	19%	38%
Cortisol	48	0.71	[0.69, 0.73]	24%	0.99	96%	29%	97%	23%	18%	0.76%	23%	58%
Hepatitis B Antigen	45	0.8	[0.73, 0.86]	2.9%	0.95	97%	-	0	100%	97%	2.9%	0	0
Hepatitis B Antigen	45	0.8	[0.73, 0.86]	2.9%	0.9	97%	-	0	100%	97%	2.9%	0	0
Hepatitis B Antigen	45	0.8	[0.73, 0.86]	2.9%	0.99	98%	60%	22%	100%	97%	2.3%	0.65%	0.43%
Hepatitis B Antigen	45	0.8	[0.73, 0.86]	2.9%	0.8	97%	-	0	100%	97%	2.9%	0	0
Iron	40	0.74	[0.71, 0.77]	40%	0.99	91%	44%	98%	17%	10%	0.95%	39%	50%

© 2019 Xu S et al. *JAMA Network Open*.

Iron	40	0.74	[0.71, 0.78]	40%	0.8	73%	64%	57%	78%	47%	17%	23%	13%
Iron	40	0.74	[0.71, 0.77]	40%	0.9	78%	53%	77%	53%	31%	9.1%	31%	28%
Iron	40	0.74	[0.71, 0.78]	40%	0.95	81%	48%	88%	36%	22%	5.0%	35%	38%
AFB Culture	40	0.68	[0.59, 0.76]	4.9%	0.99	95%	0	0	100%	95%	4.9%	0	0.10%
AFB Culture	40	0.68	[0.59, 0.76]	4.9%	0.8	95%	-	0	100%	95%	4.9%	0	0
AFB Culture	40	0.68	[0.59, 0.77]	4.9%	0.95	95%	0	0	100%	95%	4.9%	0	0.10%
AFB Culture	40	0.68	[0.60, 0.76]	4.9%	0.9	95%	-	0	100%	95%	4.9%	0	0
Calcium	39	0.86	[0.84, 0.87]	66%	0.99	89%	67%	100%	4.1%	1.4%	0.17%	66%	32%
Calcium	39	0.86	[0.84, 0.87]	66%	0.9	82%	75%	96%	36%	12%	2.7%	64%	21%
Calcium	39	0.86	[0.84, 0.87]	66%	0.8	75%	80%	91%	55%	18%	6.3%	60%	15%
Calcium	39	0.86	[0.84, 0.87]	66%	0.95	86%	70%	99%	16%	5.5%	0.93%	65%	28%
Biopsy/tissue With Gram Stain	36	0.75	[0.72, 0.79]	37%	0.8	72%	62%	42%	85%	54%	21%	16%	9.5%
Biopsy/tissue With Gram Stain	36	0.75	[0.72, 0.79]	37%	0.9	82%	54%	76%	63%	40%	8.7%	28%	24%
Biopsy/tissue With Gram Stain	36	0.75	[0.72, 0.79]	37%	0.99	88%	38%	99%	5.9%	3.8%	0.54%	36%	60%
Biopsy/tissue With Gram Stain	36	0.75	[0.71, 0.79]	37%	0.95	89%	44%	93%	32%	20%	2.4%	34%	43%
Afb Culture	35	0.81	[0.68, 0.92]	1.5%	0.8	99%	-	0	100%	99%	1.5%	0	0
Afb Culture	35	0.81	[0.66, 0.92]	1.5%	0.9	99%	-	0	100%	99%	1.5%	0	0
Afb Culture	35	0.81	[0.66, 0.92]	1.5%	0.95	99%	-	0	100%	99%	1.5%	0	0
Afb Culture	35	0.81	[0.65, 0.92]	1.5%	0.99	100%	2.4%	92%	45%	44%	0.12%	1.3%	54%
Pregnancy Test	34	0.64	[0.54, 0.74]	1.8%	0.95	98%	-	0	100%	98%	1.8%	0	0
Pregnancy Test	34	0.64	[0.54, 0.73]	1.8%	0.9	98%	-	0	100%	98%	1.8%	0	0
Pregnancy Test	34	0.64	[0.54, 0.74]	1.8%	0.8	98%	-	0	100%	98%	1.8%	0	0
Pregnancy Test	34	0.64	[0.54, 0.73]	1.8%	0.99	-	1.8%	100%	0	0	0	1.8%	98%
Urea Nitrogen	34	0.91	[0.89, 0.92]	51%	0.8	77%	89%	74%	90%	45%	13%	38%	4.7%
Urea Nitrogen	34	0.91	[0.89, 0.92]	51%	0.9	87%	82%	88%	81%	40%	6.2%	44%	9.5%
Urea Nitrogen	34	0.91	[0.89, 0.92]	51%	0.95	90%	75%	93%	68%	33%	3.7%	47%	16%
Urea Nitrogen	34	0.91	[0.89, 0.92]	51%	0.99	96%	54%	99%	13%	6.3%	0.27%	50%	43%
Gram Stain	31	0.57	[0.52, 0.63]	11%	0.9	-	11%	100%	0	0	0	11%	89%
Gram Stain	31	0.57	[0.52, 0.63]	11%	0.99	-	11%	100%	0	0	0	11%	89%
Gram Stain	31	0.57	[0.52, 0.63]	11%	0.95	-	11%	100%	0	0	0	11%	89%
Gram Stain	31	0.57	[0.52, 0.63]	11%	0.8	-	11%	100%	0	0	0	11%	89%
Teg	29	0.66	[0.61, 0.69]	54%	0.8	64%	54%	99%	3.1%	1.4%	0.80%	53%	45%
Teg	29	0.66	[0.61, 0.70]	54%	0.9	78%	54%	99%	2.4%	1.1%	0.32%	54%	45%
Teg	29	0.66	[0.61, 0.70]	54%	0.95	80%	54%	100%	1.4%	0.64%	0.16%	54%	45%
Teg	29	0.66	[0.61, 0.70]	54%	0.99	80%	54%	100%	1.4%	0.64%	0.16%	54%	45%
Protein Total	27	0.76	[0.73, 0.78]	67%	0.99	81%	67%	100%	2.6%	0.88%	0.20%	66%	33%
Protein Total	27	0.76	[0.73, 0.78]	67%	0.95	83%	68%	99%	8.1%	2.7%	0.54%	66%	31%

© 2019 Xu S et al. *JAMA Network Open*.

Protein Total	27	0.76	[0.73, 0.78]	67%	0.9	69%	71%	95%	23%	7.8%	3.6%	63%	26%
Protein Total	27	0.76	[0.73, 0.78]	67%	0.8	63%	75%	87%	44%	15%	8.7%	58%	19%
Folic Acid	26	0.63	[0.60, 0.66]	31%	0.95	80%	34%	88%	23%	16%	3.9%	28%	53%
Folic Acid	26	0.63	[0.60, 0.66]	31%	0.9	78%	39%	71%	48%	33%	9.2%	22%	35%
Folic Acid	26	0.63	[0.60, 0.66]	31%	0.8	73%	47%	31%	84%	58%	22%	9.6%	11%
Folic Acid	26	0.63	[0.60, 0.66]	31%	0.99	77%	32%	99%	1.0%	0.70%	0.21%	31%	68%
Glucose	26	0.74	[0.69, 0.79]	9.5%	0.8	91%	-	0	100%	91%	9.5%	0	0
Glucose	26	0.74	[0.69, 0.79]	9.5%	0.9	91%	-	0	100%	91%	9.5%	0	0
Glucose	26	0.74	[0.69, 0.79]	9.5%	0.95	93%	61%	28%	98%	89%	6.8%	2.6%	1.7%
Glucose	26	0.74	[0.69, 0.79]	9.5%	0.99	94%	22%	55%	79%	72%	4.3%	5.2%	19%
Respiratory Culture	24	0.67	[0.64, 0.71]	33%	0.99	82%	33%	98%	5.5%	3.7%	0.81%	32%	64%
Respiratory Culture	24	0.67	[0.64, 0.70]	33%	0.95	80%	39%	80%	38%	26%	6.4%	26%	42%
Respiratory Culture	24	0.67	[0.64, 0.70]	33%	0.9	78%	44%	64%	62%	42%	12%	21%	26%
Respiratory Culture	24	0.67	[0.64, 0.70]	33%	0.8	73%	60%	31%	90%	61%	22%	10%	6.6%
CSF Culture And Gram Stain	23	0.6	[0.48, 0.72]	3.7%	0.8	96%	-	0	100%	96%	3.7%	0	0
CSF Culture And Gram Stain	23	0.6	[0.49, 0.72]	3.7%	0.95	96%	-	0	100%	96%	3.7%	0	0
CSF Culture And Gram Stain	23	0.6	[0.48, 0.72]	3.7%	0.99	97%	10%	10%	97%	93%	3.3%	0.37%	3.3%
CSF Culture And Gram Stain	23	0.6	[0.50, 0.71]	3.7%	0.9	96%	-	0	100%	96%	3.7%	0	0
Occult Bld	23	0.76	[0.71, 0.81]	76%	0.8	70%	79%	97%	19%	4.5%	1.9%	74%	19%
Occult Bld	23	0.76	[0.71, 0.81]	76%	0.95	-	76%	100%	0	0	0	76%	24%
Occult Bld	23	0.76	[0.71, 0.81]	76%	0.99	-	76%	100%	0	0	0	76%	24%
Occult Bld	23	0.76	[0.71, 0.81]	76%	0.9	78%	77%	100%	5.1%	1.2%	0.35%	76%	23%
iSTAT Creatinine	11	0.72	[0.69, 0.75]	37%	0.8	74%	53%	59%	69%	43%	15%	22%	20%
iSTAT Creatinine	11	0.72	[0.69, 0.74]	37%	0.9	82%	46%	85%	40%	25%	5.7%	31%	37%
iSTAT Creatinine	11	0.72	[0.69, 0.75]	37%	0.95	87%	43%	94%	26%	16%	2.3%	35%	47%
iSTAT Creatinine	11	0.72	[0.69, 0.74]	37%	0.99	93%	40%	98%	14%	8.8%	0.71%	36%	54%
iSTAT Cg4	10	0.76	[0.69, 0.84]	13%	0.8	87%	-	0	100%	87%	13%	0	0
iSTAT Cg4	10	0.76	[0.68, 0.84]	13%	0.9	88%	100%	11%	100%	87%	12%	1.5%	0
iSTAT Cg4	10	0.76	[0.69, 0.84]	13%	0.95	91%	49%	42%	93%	81%	7.7%	5.6%	5.9%
iSTAT Cg4	10	0.76	[0.69, 0.84]	13%	0.99	93%	25%	67%	69%	60%	4.4%	8.9%	27%
Anti-hiv	1	0.58	[0.41, 0.75]	1.2%	0.99	99%	2.8%	44%	81%	80%	0.69%	0.54%	19%
Anti-hiv	1	0.58	[0.42, 0.74]	1.2%	0.95	99%	-	0	100%	99%	1.2%	0	0
Anti-hiv	1	0.58	[0.43, 0.75]	1.2%	0.9	99%	-	0	100%	99%	1.2%	0	0
Anti-hiv	1	0.58	[0.41, 0.74]	1.2%	0.8	99%	-	0	100%	99%	1.2%	0	0
Stool Culture	0	0.68	[0.57, 0.79]	9.0%	0.99	100%	9.3%	100%	3.4%	3.1%	0	9.0%	88%
Stool Culture	0	0.68	[0.57, 0.79]	9.0%	0.95	95%	11%	78%	38%	34%	2.0%	7.0%	57%
Stool Culture	0	0.68	[0.57, 0.79]	9.0%	0.9	92%	50%	8.7%	99%	90%	8.2%	0.78%	0.78%

© 2019 Xu S et al. *JAMA Network Open*.

Stool Culture	0	0.68	[0.57, 0.80]	9.0%	0.8	91%	-	0	100%	91%	9.0%	0	0

eTable 3. Diagnostic Metrics for Common Stanford Standalone Labs

Lab Test	Vol	AUROC	95% CI	Prev	Target NPV	NPV	PPV	Sens	Spec	TN	FN	TP	FP
Potassium	9546	0.76	[0.73, 0.79]	13%	0.99	98%	13%	100%	2.5%	2.2%	0.04%	13%	85%
Potassium	9546	0.76	[0.73, 0.79]	13%	0.8	87%	-	0	100%	87%	13%	0	0
Potassium	9546	0.76	[0.73, 0.79]	13%	0.9	89%	48%	19%	97%	84%	11%	2.4%	2.6%
Potassium	9546	0.76	[0.73, 0.79]	13%	0.95	95%	25%	77%	66%	57%	3.0%	10%	30%
Hemoglobin	8016	0.94	[0.92, 0.95]	88%	0.99	85%	89%	100%	7.9%	0.93%	0.17%	88%	11%
Hemoglobin	8016	0.94	[0.92, 0.95]	88%	0.95	81%	90%	99%	17%	2.1%	0.50%	88%	9.8%
Hemoglobin	8016	0.94	[0.92, 0.95]	88%	0.9	77%	92%	99%	36%	4.3%	1.3%	87%	7.6%
Hemoglobin	8016	0.94	[0.92, 0.95]	88%	0.8	69%	95%	96%	59%	6.9%	3.2%	85%	4.9%
Sodium	7289	0.87	[0.85, 0.88]	42%	0.99	98%	42%	100%	3.0%	1.7%	0.04%	42%	57%
Sodium	7289	0.87	[0.85, 0.88]	42%	0.8	75%	87%	56%	94%	55%	18%	23%	3.5%
Sodium	7289	0.87	[0.85, 0.88]	42%	0.9	86%	68%	84%	71%	41%	6.5%	35%	17%
Sodium	7289	0.87	[0.85, 0.88]	42%	0.95	92%	53%	95%	41%	24%	2.1%	40%	34%
Creatinine	7012	0.96	[0.96, 0.97]	40%	0.99	99%	63%	99%	61%	36%	0.49%	40%	23%
Creatinine	7012	0.96	[0.96, 0.97]	40%	0.95	94%	79%	93%	83%	50%	2.9%	37%	10.0%
Creatinine	7012	0.96	[0.96, 0.97]	40%	0.9	90%	89%	85%	93%	56%	6.0%	34%	4.1%
Creatinine	7012	0.96	[0.96, 0.97]	40%	0.8	80%	98%	64%	99%	59%	15%	26%	0.56%
Urea Nitrogen	6999	0.95	[0.94, 0.96]	30%	0.8	81%	96%	45%	99%	69%	16%	13%	0.63%
Urea Nitrogen	6999	0.95	[0.94, 0.96]	30%	0.9	89%	90%	73%	97%	68%	8.0%	22%	2.4%
Urea Nitrogen	6999	0.95	[0.94, 0.96]	30%	0.99	99%	42%	99%	41%	29%	0.27%	30%	41%
Urea Nitrogen	6999	0.95	[0.93, 0.96]	30%	0.95	94%	77%	87%	89%	62%	4.0%	26%	7.9%
Calcium	6963	0.89	[0.87, 0.90]	59%	0.99	100%	60%	100%	3.9%	1.6%	0	59%	39%
Calcium	6963	0.89	[0.87, 0.90]	59%	0.95	92%	65%	99%	24%	9.8%	0.80%	58%	31%
Calcium	6963	0.89	[0.87, 0.90]	59%	0.9	88%	71%	96%	45%	18%	2.6%	57%	23%
Calcium	6963	0.89	[0.87, 0.90]	59%	0.8	79%	82%	87%	73%	30%	7.9%	51%	11%
CO2	6929	0.86	[0.84, 0.88]	19%	0.99	95%	35%	88%	60%	48%	2.3%	17%	32%
CO2	6929	0.86	[0.84, 0.88]	19%	0.95	93%	54%	72%	85%	69%	5.5%	14%	12%
CO2	6929	0.86	[0.85, 0.88]	19%	0.9	88%	83%	47%	98%	79%	10%	9.0%	1.9%
CO2	6929	0.86	[0.84, 0.88]	19%	0.8	81%	-	0	100%	81%	19%	0	0
Platelet Count	6660	0.91	[0.90, 0.92]	45%	0.8	76%	88%	64%	93%	51%	16%	29%	3.8%
Platelet Count	6660	0.91	[0.90, 0.92]	45%	0.9	89%	80%	88%	82%	45%	5.6%	40%	9.8%
Platelet Count	6660	0.91	[0.90, 0.92]	45%	0.95	92%	72%	93%	70%	39%	3.1%	42%	16%
Platelet Count	6660	0.91	[0.90, 0.92]	45%	0.99	98%	59%	99%	42%	23%	0.38%	45%	32%

White Blood Cells	6422	0.89	[0.88, 0.91]	45%	0.99	100%	45%	100%	0.54%	0.30%	0	45%	55%
White Blood Cells	6422	0.89	[0.88, 0.91]	45%	0.95	93%	60%	95%	48%	27%	2.1%	42%	29%
White Blood Cells	6422	0.89	[0.88, 0.90]	45%	0.9	87%	70%	87%	70%	39%	5.9%	39%	16%
White Blood Cells	6422	0.89	[0.88, 0.91]	45%	0.8	78%	88%	67%	93%	51%	15%	30%	4.0%
Albumin	3063	0.93	[0.91, 0.94]	84%	0.99	-	84%	100%	0	0	0	84%	16%
Albumin	3063	0.93	[0.91, 0.94]	84%	0.8	-	84%	100%	0	0	0	84%	16%
Albumin	3063	0.93	[0.91, 0.94]	84%	0.9	-	84%	100%	0	0	0	84%	16%
Albumin	3063	0.93	[0.91, 0.94]	84%	0.95	-	84%	100%	0	0	0	84%	16%
Total Bilirubin	3038	0.97	[0.96, 0.98]	29%	0.9	87%	99%	65%	100%	71%	10%	19%	0.14%
Total Bilirubin	3038	0.97	[0.96, 0.97]	29%	0.8	74%	100%	14%	100%	71%	25%	4.0%	0
Total Bilirubin	3038	0.97	[0.96, 0.98]	29%	0.99	98%	58%	96%	72%	51%	1.1%	28%	20%
Total Bilirubin	3038	0.97	[0.96, 0.98]	29%	0.95	94%	92%	84%	97%	69%	4.8%	24%	2.0%
Protein	2993	0.91	[0.89, 0.92]	31%	0.8	83%	90%	57%	97%	67%	13%	18%	2.0%
Protein	2993	0.91	[0.89, 0.92]	31%	0.9	90%	74%	78%	88%	60%	6.8%	24%	8.6%
Protein	2993	0.91	[0.89, 0.92]	31%	0.95	93%	59%	89%	72%	50%	3.5%	28%	19%
Protein	2993	0.91	[0.90, 0.92]	31%	0.99	97%	39%	98%	31%	21%	0.72%	31%	48%
AST (SGOT)	2986	0.92	[0.91, 0.93]	35%	0.95	94%	61%	92%	69%	45%	2.8%	32%	21%
AST (SGOT)	2986	0.92	[0.91, 0.93]	35%	0.99	97%	44%	98%	34%	22%	0.70%	34%	43%
AST (SGOT)	2986	0.92	[0.91, 0.93]	35%	0.9	89%	78%	80%	88%	57%	6.8%	28%	8.0%
AST (SGOT)	2986	0.92	[0.91, 0.93]	35%	0.8	81%	96%	57%	99%	65%	15%	20%	0.83%
ALT (SGPT)	2986	0.93	[0.92, 0.94]	30%	0.99	98%	45%	98%	47%	33%	0.74%	30%	37%
ALT (SGPT)	2986	0.93	[0.92, 0.94]	30%	0.95	93%	71%	85%	85%	59%	4.7%	26%	11%
ALT (SGPT)	2986	0.93	[0.92, 0.94]	30%	0.9	89%	94%	71%	98%	68%	8.8%	22%	1.5%
ALT (SGPT)	2986	0.93	[0.92, 0.94]	30%	0.8	76%	99%	30%	100%	69%	21%	9.0%	0.12%
Alk Phos	2984	0.94	[0.93, 0.95]	45%	0.8	69%	99%	45%	100%	55%	25%	20%	0.22%
Alk Phos	2984	0.94	[0.93, 0.95]	45%	0.95	90%	77%	89%	78%	43%	4.8%	40%	12%
Alk Phos	2984	0.94	[0.93, 0.95]	45%	0.99	97%	60%	98%	47%	26%	0.84%	44%	29%
Alk Phos	2984	0.94	[0.93, 0.95]	45%	0.9	86%	91%	81%	93%	52%	8.7%	36%	3.7%

eTable 4. Diagnostic Metrics for Common Stanford Components

Lab Test	AUROC	95% CI	Prev	Target NPV	NPV	PPV	Sens	Spec	TN	FN	TP	FP
Magnesium	0.83	[0.80, 0.86]	9.1%	0.99	97%	16%	81%	59%	53%	1.7%	7.4%	38%
Magnesium	0.83	[0.79, 0.86]	9.1%	0.95	94%	61%	41%	97%	89%	5.4%	3.7%	2.3%
Magnesium	0.83	[0.80, 0.86]	9.1%	0.9	91%	-	0	100%	91%	9.1%	0	0
Magnesium	0.83	[0.79, 0.86]	9.1%	0.8	91%	-	0	100%	91%	9.1%	0	0

© 2019 Xu S et al. *JAMA Network Open*.

Phosphorus	0.75	[0.73, 0.77]	31%	0.99	95%	33%	99%	6.5%	4.5%	0.23%	31%	64%
Phosphorus	0.75	[0.73, 0.78]	31%	0.95	90%	36%	94%	25%	17%	2.0%	29%	51%
Phosphorus	0.75	[0.73, 0.78]	31%	0.9	86%	43%	81%	52%	35%	5.9%	25%	33%
Phosphorus	0.75	[0.73, 0.77]	31%	0.8	78%	62%	46%	87%	60%	17%	15%	8.9%
Hemoglobin A1c	0.69	[0.64, 0.73]	71%	0.99	87%	72%	100%	6.9%	2.0%	0.31%	70%	27%
Hemoglobin A1c	0.69	[0.64, 0.73]	71%	0.95	57%	74%	94%	18%	5.3%	4.0%	67%	24%
Hemoglobin A1c	0.69	[0.64, 0.73]	71%	0.9	52%	74%	91%	24%	7.0%	6.3%	64%	22%
Hemoglobin A1c	0.69	[0.64, 0.73]	71%	0.8	50%	77%	84%	39%	11%	11%	59%	18%
Uric Acid	0.88	[0.84, 0.92]	38%	0.99	96%	45%	98%	25%	16%	0.64%	38%	46%
Uric Acid	0.88	[0.84, 0.92]	38%	0.95	92%	63%	91%	67%	41%	3.5%	35%	20%
Uric Acid	0.88	[0.84, 0.92]	38%	0.9	86%	72%	79%	81%	50%	8.0%	30%	12%
Uric Acid	0.88	[0.84, 0.92]	38%	0.8	80%	88%	61%	95%	58%	15%	23%	3.2%
Albumin	0.76	[0.65, 0.86]	69%	0.99	50%	70%	96%	8.3%	2.6%	2.6%	66%	29%
Albumin	0.76	[0.65, 0.86]	69%	0.95	50%	72%	89%	25%	7.8%	7.8%	61%	23%
Albumin	0.76	[0.65, 0.86]	69%	0.9	54%	79%	79%	54%	17%	14%	55%	14%
Albumin	0.76	[0.66, 0.86]	69%	0.8	45%	84%	58%	75%	23%	29%	40%	7.8%
Thyroid Stimulating Hormone	0.67	[0.63, 0.70]	15%	0.99	92%	17%	87%	27%	23%	1.9%	13%	62%
Thyroid Stimulating Hormone	0.67	[0.63, 0.71]	15%	0.95	89%	24%	46%	75%	64%	8.0%	6.7%	21%
Thyroid Stimulating Hormone	0.67	[0.62, 0.71]	15%	0.9	88%	47%	24%	95%	81%	11%	3.6%	4.1%
Thyroid Stimulating Hormone	0.67	[0.63, 0.71]	15%	0.8	85%	-	0	100%	85%	15%	0	0
Troponin I	0.95	[0.94, 0.96]	32%	0.99	96%	62%	94%	73%	50%	1.8%	30%	18%
Troponin I	0.95	[0.94, 0.96]	32%	0.95	93%	80%	85%	90%	61%	4.9%	27%	7.0%
Troponin I	0.95	[0.94, 0.96]	32%	0.9	90%	90%	77%	96%	65%	7.5%	25%	2.7%
Troponin I	0.95	[0.94, 0.96]	32%	0.8	80%	100%	46%	100%	68%	17%	15%	0
Potassium	0.7	[0.63, 0.78]	38%	0.99	84%	45%	90%	33%	21%	3.9%	34%	42%
Potassium	0.7	[0.62, 0.78]	38%	0.95	76%	52%	69%	61%	38%	12%	26%	24%
Potassium	0.7	[0.62, 0.77]	38%	0.9	74%	60%	55%	77%	48%	17%	21%	14%
Potassium	0.7	[0.62, 0.78]	38%	0.8	68%	66%	28%	91%	57%	27%	11%	5.6%
Sodium	0.94	[0.90, 0.98]	51%	0.99	86%	94%	85%	95%	46%	7.6%	43%	2.5%
Sodium	0.94	[0.90, 0.98]	51%	0.95	83%	98%	80%	99%	48%	10%	41%	0.64%
Sodium	0.94	[0.90, 0.98]	51%	0.9	72%	98%	64%	99%	48%	18%	32%	0.64%
Sodium	0.94	[0.90, 0.98]	51%	0.8	59%	97%	35%	99%	48%	33%	18%	0.64%
Calcium	0.81	[0.69, 0.91]	63%	0.99	64%	95%	69%	93%	34%	20%	44%	2.4%
Calcium	0.81	[0.71, 0.91]	63%	0.95	64%	95%	69%	93%	34%	20%	44%	2.4%
Calcium	0.81	[0.69, 0.91]	63%	0.9	64%	95%	69%	93%	34%	20%	44%	2.4%
Calcium	0.81	[0.71, 0.91]	63%	0.8	64%	95%	69%	93%	34%	20%	44%	2.4%

© 2019 Xu S et al. *JAMA Network Open*.

eTable 5. Diagnostic Metrics for Top UMich Standalone Labs

Lab Test	AUROC	95% CI	Prev	Target NPV	NPV	PPV	Sens	Spec	TN	FN	TP	FP
White Blood Cells	0.83	[0.81, 0.84]	46%	0.99	94%	51%	99%	19%	10%	0.66%	45%	44%
White Blood Cells	0.83	[0.81, 0.84]	46%	0.95	89%	55%	95%	34%	18%	2.2%	44%	36%
White Blood Cells	0.83	[0.81, 0.85]	46%	0.9	84%	61%	88%	53%	28%	5.6%	41%	25%
White Blood Cells	0.83	[0.81, 0.85]	46%	0.8	75%	74%	70%	78%	42%	14%	32%	12%
Hemoglobin	0.94	[0.93, 0.95]	77%	0.99	85%	79%	99%	14%	3.2%	0.58%	76%	20%
Hemoglobin	0.94	[0.93, 0.95]	77%	0.95	82%	81%	99%	22%	5.0%	1.1%	76%	18%
Hemoglobin	0.94	[0.93, 0.95]	77%	0.9	80%	83%	98%	33%	7.7%	1.9%	75%	15%
Hemoglobin	0.94	[0.93, 0.95]	77%	0.8	73%	89%	93%	62%	14%	5.2%	72%	8.9%
Platelet Count	0.92	[0.90, 0.93]	32%	0.99	98%	41%	98%	34%	23%	0.48%	31%	45%
Platelet Count	0.92	[0.91, 0.93]	32%	0.95	93%	66%	88%	79%	54%	3.9%	28%	14%
Platelet Count	0.92	[0.90, 0.93]	32%	0.9	89%	83%	74%	93%	64%	8.1%	23%	4.6%
Platelet Count	0.92	[0.90, 0.93]	32%	0.8	79%	98%	41%	100%	68%	18%	13%	0.29%
Sodium	0.92	[0.90, 0.93]	22%	0.99	-	22%	100%	0	0	0	22%	78%
Sodium	0.92	[0.90, 0.93]	22%	0.95	96%	50%	88%	76%	60%	2.5%	19%	19%
Sodium	0.92	[0.90, 0.93]	22%	0.9	93%	84%	72%	96%	76%	6.1%	15%	2.9%
Sodium	0.92	[0.90, 0.93]	22%	0.8	78%	-	0	100%	78%	22%	0	0
Potassium	0.76	[0.74, 0.79]	13%	0.99	100%	13%	100%	2.1%	1.8%	0	13%	85%
Potassium	0.76	[0.74, 0.79]	13%	0.95	95%	21%	83%	54%	47%	2.3%	11%	40%
Potassium	0.76	[0.74, 0.79]	13%	0.9	91%	40%	36%	92%	80%	8.3%	4.8%	7.0%
Potassium	0.76	[0.74, 0.79]	13%	0.8	87%	-	0	100%	87%	13%	0	0
Creatinine	0.9	[0.89, 0.91]	41%	0.99	95%	50%	97%	32%	19%	1.0%	40%	40%
Creatinine	0.9	[0.89, 0.91]	41%	0.95	91%	63%	91%	62%	36%	3.7%	38%	22%
Creatinine	0.9	[0.88, 0.91]	41%	0.9	87%	77%	82%	83%	48%	7.4%	34%	10%
Creatinine	0.9	[0.89, 0.91]	41%	0.8	77%	92%	60%	96%	56%	16%	25%	2.2%
Total Bilirubin	0.93	[0.91, 0.94]	28%	0.99	94%	63%	86%	81%	58%	3.9%	24%	14%
Total Bilirubin	0.93	[0.91, 0.94]	28%	0.95	92%	87%	79%	96%	69%	5.8%	22%	3.1%
Total Bilirubin	0.93	[0.91, 0.94]	28%	0.9	88%	99%	65%	100%	72%	9.7%	18%	0.23%
Total Bilirubin	0.93	[0.91, 0.94]	28%	0.8	77%	100%	22%	100%	72%	22%	6.0%	0
CO2	0.87	[0.84, 0.89]	15%	0.99	100%	15%	100%	0.37%	0.31%	0	15%	85%
CO2	0.87	[0.84, 0.89]	15%	0.95	94%	52%	68%	89%	76%	4.9%	10%	9.3%
CO2	0.87	[0.85, 0.89]	15%	0.9	88%	83%	24%	99%	84%	11%	3.7%	0.74%

© 2019 Xu S et al. *JAMA Network Open*.

CO2	0.87	[0.85, 0.89]	15%	0.8	85%	-	0	100%	85%	15%	0	0
AST (SGOT)	0.88	[0.86, 0.89]	48%	0.99	95%	50%	100%	6.8%	3.5%	0.20%	48%	48%
AST (SGOT)	0.88	[0.87, 0.89]	48%	0.95	93%	56%	98%	29%	15%	1.1%	47%	37%
AST (SGOT)	0.88	[0.87, 0.89]	48%	0.9	90%	61%	94%	45%	23%	2.6%	45%	28%
AST (SGOT)	0.88	[0.87, 0.89]	48%	0.8	79%	79%	77%	81%	42%	11%	37%	9.8%
ALT (SGPT)	0.92	[0.91, 0.93]	40%	0.99	99%	45%	100%	21%	13%	0.19%	40%	48%
ALT (SGPT)	0.92	[0.91, 0.93]	40%	0.95	96%	59%	96%	56%	34%	1.5%	38%	26%
ALT (SGPT)	0.92	[0.91, 0.93]	40%	0.9	90%	68%	88%	73%	44%	4.7%	35%	17%
ALT (SGPT)	0.92	[0.91, 0.93]	40%	0.8	82%	95%	68%	97%	59%	13%	27%	1.6%
Albumin	0.9	[0.89, 0.92]	43%	0.99	97%	49%	99%	20%	12%	0.32%	43%	45%
Albumin	0.9	[0.89, 0.92]	43%	0.95	93%	56%	96%	43%	24%	1.9%	42%	32%
Albumin	0.9	[0.89, 0.92]	43%	0.9	87%	73%	86%	75%	43%	6.3%	37%	14%
Albumin	0.9	[0.89, 0.92]	43%	0.8	77%	93%	62%	96%	55%	16%	27%	2.0%
Calcium	0.89	[0.88, 0.90]	35%	0.99	98%	40%	99%	20%	13%	0.20%	35%	52%
Calcium	0.89	[0.88, 0.90]	35%	0.95	93%	55%	92%	59%	39%	2.8%	32%	27%
Calcium	0.89	[0.88, 0.90]	35%	0.9	89%	68%	81%	80%	52%	6.7%	28%	13%
Calcium	0.89	[0.88, 0.90]	35%	0.8	81%	88%	57%	96%	62%	15%	20%	2.8%
Protein	0.91	[0.90, 0.92]	43%	0.99	99%	46%	100%	11%	6.0%	0.07%	43%	51%
Protein	0.91	[0.90, 0.92]	43%	0.95	93%	55%	96%	39%	22%	1.6%	42%	34%
Protein	0.91	[0.90, 0.92]	43%	0.9	89%	76%	87%	80%	45%	5.5%	38%	12%
Protein	0.91	[0.90, 0.92]	43%	0.8	79%	94%	66%	97%	55%	15%	28%	1.9%
Alk Phos	0.92	[0.91, 0.93]	27%	0.99	99%	39%	99%	43%	31%	0.27%	27%	42%
Alk Phos	0.92	[0.91, 0.93]	27%	0.95	94%	58%	87%	77%	56%	3.5%	24%	17%
Alk Phos	0.92	[0.91, 0.93]	27%	0.9	90%	83%	71%	94%	69%	7.8%	20%	4.0%
Alk Phos	0.92	[0.91, 0.93]	27%	0.8	83%	99%	48%	100%	73%	14%	13%	0.12%
Urea Nitrogen	0.93	[0.92, 0.94]	48%	0.99	97%	55%	99%	26%	13%	0.41%	48%	39%
Urea Nitrogen	0.93	[0.92, 0.94]	48%	0.95	93%	69%	95%	60%	31%	2.2%	46%	21%
Urea Nitrogen	0.93	[0.92, 0.94]	48%	0.9	89%	81%	90%	80%	42%	5.0%	43%	10%
Urea Nitrogen	0.93	[0.92, 0.94]	48%	0.8	78%	94%	71%	96%	50%	14%	34%	2.3%

eTable 6. Diagnostic Metrics for Common UMich Components

Lab Test	AUROC	95% CI	Prev	Target NPV	NPV	PPV	Sens	Spec	TN	FN	TP	FP
Magnesium	0.81	[0.79, 0.83]	21%	0.99	-	21%	100%	0	0	0	21%	79%
Magnesium	0.81	[0.78, 0.83]	21%	0.95	95%	32%	90%	48%	37%	2.1%	19%	41%
Magnesium	0.81	[0.78, 0.83]	21%	0.9	90%	42%	71%	73%	58%	6.1%	15%	21%
Magnesium	0.81	[0.79, 0.83]	21%	0.8	82%	79%	21%	98%	77%	17%	4.5%	1.2%
Phosphorus	0.8	[0.78, 0.82]	24%	0.99	97%	26%	99%	9.4%	7.1%	0.24%	24%	69%
Phosphorus	0.8	[0.78, 0.82]	24%	0.95	91%	39%	81%	60%	46%	4.5%	20%	30%

© 2019 Xu S et al. *JAMA Network Open*.

Phosphorus	0.8	[0.78, 0.82]	24%	0.9	88%	55%	62%	84%	64%	9.0%	15%	12%
Phosphorus	0.8	[0.78, 0.82]	24%	0.8	79%	88%	15%	99%	75%	20%	3.7%	0.52%
Prothrombin Time	0.93	[0.92, 0.94]	44%	0.99	97%	50%	99%	24%	13%	0.40%	44%	43%
Prothrombin Time	0.93	[0.92, 0.94]	44%	0.95	92%	69%	93%	67%	38%	3.2%	41%	18%
Prothrombin Time	0.93	[0.92, 0.94]	44%	0.9	87%	83%	84%	87%	49%	7.1%	37%	7.5%
Prothrombin Time	0.93	[0.92, 0.94]	44%	0.8	78%	96%	66%	98%	55%	15%	29%	1.1%
Partial Thromboplastin Time	0.92	[0.91, 0.93]	49%	0.99	93%	60%	97%	38%	20%	1.6%	48%	31%
Partial Thromboplastin Time	0.92	[0.91, 0.93]	49%	0.95	89%	76%	91%	72%	36%	4.5%	45%	14%
Partial Thromboplastin Time	0.92	[0.91, 0.93]	49%	0.9	86%	84%	86%	84%	43%	7.0%	42%	8.1%
Partial Thromboplastin Time	0.92	[0.91, 0.93]	49%	0.8	81%	94%	77%	95%	48%	11%	38%	2.4%
Alkaline Phosphatase	0.9	[0.89, 0.91]	46%	0.99	96%	55%	99%	30%	16%	0.64%	46%	37%
Alkaline Phosphatase	0.9	[0.89, 0.91]	46%	0.95	93%	63%	95%	51%	27%	2.2%	44%	26%
Alkaline Phosphatase	0.9	[0.89, 0.91]	46%	0.9	85%	72%	85%	71%	38%	6.8%	40%	16%
Alkaline Phosphatase	0.9	[0.89, 0.91]	46%	0.8	78%	90%	70%	93%	50%	14%	32%	3.8%
Sodium	0.87	[0.86, 0.89]	41%	0.99	-	41%	100%	0	0	0	41%	59%
Sodium	0.87	[0.86, 0.89]	41%	0.95	92%	55%	94%	45%	26%	2.3%	39%	32%
Sodium	0.87	[0.86, 0.89]	41%	0.9	87%	64%	86%	66%	39%	5.7%	36%	20%
Sodium	0.87	[0.86, 0.89]	41%	0.8	78%	86%	62%	93%	54%	16%	26%	4.3%
Potassium	0.76	[0.74, 0.79]	17%	0.99	96%	22%	94%	31%	26%	1.1%	16%	57%
Potassium	0.76	[0.73, 0.79]	17%	0.95	92%	30%	71%	66%	55%	4.9%	12%	28%
Potassium	0.76	[0.73, 0.79]	17%	0.9	88%	44%	37%	91%	75%	11%	6.2%	7.8%
Potassium	0.76	[0.74, 0.79]	17%	0.8	83%	-	0	100%	83%	17%	0	0
Troponin I	0.89	[0.88, 0.91]	44%	0.99	96%	48%	99%	15%	8.6%	0.36%	44%	47%
Troponin I	0.89	[0.88, 0.91]	44%	0.95	91%	61%	94%	52%	29%	2.8%	42%	27%
Troponin I	0.89	[0.88, 0.91]	44%	0.9	86%	71%	86%	72%	40%	6.3%	38%	16%
Troponin I	0.89	[0.88, 0.91]	44%	0.8	80%	86%	72%	91%	50%	12%	32%	5.2%
Lactate Dehydrogenase	0.94	[0.93, 0.95]	66%	0.99	97%	72%	100%	25%	8.2%	0.27%	66%	25%
Lactate Dehydrogenase	0.94	[0.93, 0.95]	66%	0.95	94%	78%	99%	45%	15%	0.97%	65%	18%
Lactate Dehydrogenase	0.94	[0.93, 0.95]	66%	0.9	87%	82%	96%	58%	20%	2.8%	64%	14%
Lactate Dehydrogenase	0.94	[0.93, 0.95]	66%	0.8	76%	90%	87%	81%	27%	8.5%	58%	6.2%
Calcium, Ionized	0.86	[0.84, 0.87]	63%	0.99	92%	64%	100%	3.7%	1.4%	0.11%	63%	36%
Calcium, Ionized	0.86	[0.84, 0.87]	63%	0.95	89%	69%	98%	24%	9.0%	1.2%	62%	28%
Calcium, Ionized	0.86	[0.84, 0.87]	63%	0.9	83%	75%	95%	45%	17%	3.4%	60%	20%
Calcium, Ionized	0.86	[0.85, 0.87]	63%	0.8	74%	85%	84%	75%	28%	9.9%	53%	9.2%
Uric Acid	0.91	[0.90, 0.93]	46%	0.99	97%	54%	99%	30%	17%	0.49%	45%	38%
Uric Acid	0.91	[0.90, 0.93]	46%	0.95	94%	61%	97%	48%	26%	1.5%	44%	28%
Uric Acid	0.91	[0.90, 0.93]	46%	0.9	90%	71%	90%	70%	38%	4.4%	41%	16%
Uric Acid	0.91	[0.89, 0.93]	46%	0.8	83%	84%	79%	88%	48%	9.7%	36%	6.7%
Albumin	0.85	[0.82, 0.87]	79%	0.99	100%	80%	100%	3.5%	0.73%	0	79%	20%

© 2019 Xu S et al. *JAMA Network Open*.

Albumin	0.85	[0.82, 0.87]	79%	0.95	54%	80%	99%	6.6%	1.4%	1.2%	78%	19%
Albumin	0.85	[0.82, 0.87]	79%	0.9	58%	82%	97%	18%	3.8%	2.7%	77%	17%
Albumin	0.85	[0.82, 0.87]	79%	0.8	56%	85%	92%	40%	8.3%	6.4%	73%	12%
Thyroid Stimulating Hormone	0.66	[0.61, 0.71]	33%	0.99	80%	36%	88%	23%	15%	3.9%	29%	52%
Thyroid Stimulating Hormone	0.66	[0.61, 0.71]	33%	0.95	76%	42%	65%	56%	37%	12%	21%	30%
Thyroid Stimulating Hormone	0.66	[0.61, 0.71]	33%	0.9	74%	50%	42%	79%	53%	19%	14%	14%
Thyroid Stimulating Hormone	0.66	[0.61, 0.71]	33%	0.8	71%	65%	19%	95%	64%	26%	6.4%	3.5%

eTable 7. Diagnostic Metrics for Top UCSF Standalone Labs

Lab Test	AUROC	95% CI	Prev	Target NPV	NPV	PPV	Sens	Spec	TN	FN	TP	FP
White Blood Cells	0.89	[0.87, 0.90]	52%	0.99	94%	54%	99%	8.4%	4.0%	0.28%	51%	44%
White Blood Cells	0.89	[0.87, 0.90]	52%	0.95	88%	63%	95%	41%	20%	2.6%	49%	28%
White Blood Cells	0.89	[0.87, 0.90]	52%	0.9	85%	71%	90%	60%	29%	5.0%	47%	19%
White Blood Cells	0.89	[0.87, 0.90]	52%	0.8	76%	86%	74%	88%	42%	14%	38%	6.0%
Hemoglobin	0.95	[0.94, 0.96]	89%	0.99	81%	90%	100%	4.3%	0.46%	0.11%	89%	10%
Hemoglobin	0.95	[0.93, 0.96]	89%	0.95	76%	90%	100%	11%	1.1%	0.35%	89%	9.4%
Hemoglobin	0.95	[0.94, 0.96]	89%	0.9	82%	92%	99%	25%	2.7%	0.60%	89%	7.8%
Hemoglobin	0.95	[0.94, 0.96]	89%	0.8	73%	93%	98%	40%	4.3%	1.6%	88%	6.3%
Platelet Count	0.95	[0.94, 0.96]	39%	0.99	97%	60%	98%	57%	35%	0.98%	38%	26%
Platelet Count	0.95	[0.94, 0.96]	39%	0.95	92%	80%	89%	85%	52%	4.4%	35%	8.9%
Platelet Count	0.95	[0.94, 0.96]	39%	0.9	88%	92%	79%	96%	58%	8.1%	31%	2.6%
Platelet Count	0.95	[0.94, 0.96]	39%	0.8	76%	99%	50%	100%	60%	20%	20%	0.20%
Sodium	0.89	[0.87, 0.90]	33%	0.99	100%	33%	100%	0.55%	0.37%	0	33%	67%
Sodium	0.89	[0.88, 0.90]	33%	0.95	95%	49%	95%	53%	35%	1.7%	31%	32%
Sodium	0.89	[0.88, 0.90]	33%	0.9	90%	64%	81%	78%	53%	6.1%	26%	15%
Sodium	0.89	[0.87, 0.90]	33%	0.8	82%	86%	56%	96%	64%	14%	18%	3.0%
Potassium	0.77	[0.75, 0.79]	16%	0.99	97%	20%	95%	28%	23%	0.84%	15%	61%
Potassium	0.77	[0.74, 0.79]	16%	0.95	94%	28%	79%	61%	51%	3.4%	13%	33%
Potassium	0.77	[0.74, 0.79]	16%	0.9	90%	41%	49%	87%	73%	8.3%	7.8%	11%
Potassium	0.77	[0.74, 0.79]	16%	0.8	84%	-	0	100%	84%	16%	0	0
Creatinine	0.94	[0.93, 0.95]	41%	0.99	96%	47%	99%	23%	14%	0.51%	40%	45%
Creatinine	0.94	[0.93, 0.95]	41%	0.95	93%	72%	92%	76%	45%	3.2%	37%	14%
Creatinine	0.94	[0.93, 0.95]	41%	0.9	89%	91%	83%	94%	56%	6.9%	34%	3.4%
Creatinine	0.94	[0.93, 0.95]	41%	0.8	75%	98%	52%	99%	59%	19%	21%	0.47%
Total Bilirubin	0.93	[0.91, 0.94]	30%	0.99	98%	44%	98%	48%	34%	0.70%	29%	37%

© 2019 Xu S et al. *JAMA Network Open*.

Total Bilirubin	0.93	[0.91, 0.94]	30%	0.95	93%	64%	87%	80%	56%	3.9%	26%	14%
Total Bilirubin	0.93	[0.91, 0.94]	30%	0.9	89%	85%	72%	95%	67%	8.4%	21%	3.7%
Total Bilirubin	0.93	[0.91, 0.94]	30%	0.8	81%	99%	45%	100%	70%	16%	13%	0.12%
CO2	0.87	[0.86, 0.89]	21%	0.99	97%	24%	98%	13%	11%	0.37%	21%	68%
CO2	0.87	[0.86, 0.89]	21%	0.95	95%	43%	86%	69%	55%	3.0%	18%	24%
CO2	0.87	[0.86, 0.89]	21%	0.9	90%	67%	62%	92%	72%	8.1%	13%	6.6%
CO2	0.87	[0.86, 0.89]	21%	0.8	79%	91%	1.7%	100%	79%	21%	0.37%	0.04%
AST (SGOT)	0.85	[0.83, 0.86]	46%	0.99	94%	47%	100%	4.8%	2.6%	0.17%	46%	52%
AST (SGOT)	0.85	[0.83, 0.86]	46%	0.95	90%	54%	96%	30%	16%	1.9%	44%	38%
AST (SGOT)	0.85	[0.83, 0.86]	46%	0.9	86%	61%	90%	51%	28%	4.5%	41%	27%
AST (SGOT)	0.85	[0.83, 0.86]	46%	0.8	77%	73%	72%	78%	42%	13%	33%	12%
ALT (SGPT)	0.91	[0.90, 0.93]	31%	0.99	99%	41%	99%	36%	25%	0.35%	31%	44%
ALT (SGPT)	0.91	[0.90, 0.93]	31%	0.95	93%	56%	89%	67%	46%	3.4%	28%	22%
ALT (SGPT)	0.91	[0.90, 0.93]	31%	0.9	89%	80%	76%	91%	63%	7.7%	24%	6.0%
ALT (SGPT)	0.91	[0.90, 0.93]	31%	0.8	78%	99%	39%	100%	69%	19%	12%	0.12%
Albumin	0.91	[0.90, 0.92]	78%	0.99	100%	78%	100%	0.18%	0.04%	0	78%	22%
Albumin	0.91	[0.90, 0.92]	78%	0.95	100%	78%	100%	0.18%	0.04%	0	78%	22%
Albumin	0.91	[0.90, 0.92]	78%	0.9	79%	80%	99%	9.1%	2.0%	0.52%	78%	20%
Albumin	0.91	[0.90, 0.92]	78%	0.8	75%	83%	97%	31%	6.8%	2.2%	76%	15%
Calcium	0.88	[0.87, 0.89]	69%	0.99	88%	71%	99%	10%	3.2%	0.42%	69%	28%
Calcium	0.88	[0.87, 0.90]	69%	0.95	88%	74%	99%	23%	7.2%	0.96%	68%	24%
Calcium	0.88	[0.87, 0.90]	69%	0.9	85%	78%	97%	39%	12%	2.1%	67%	19%
Calcium	0.88	[0.87, 0.90]	69%	0.8	75%	83%	91%	59%	18%	6.1%	63%	13%
Protein	0.9	[0.89, 0.91]	54%	0.99	97%	57%	100%	12%	5.5%	0.16%	54%	40%
Protein	0.9	[0.89, 0.91]	54%	0.95	93%	62%	98%	30%	14%	1.1%	53%	32%
Protein	0.9	[0.89, 0.91]	54%	0.9	88%	69%	94%	51%	24%	3.2%	51%	22%
Protein	0.9	[0.89, 0.91]	54%	0.8	81%	84%	83%	81%	37%	9.0%	45%	8.7%
Alk Phos	0.93	[0.92, 0.94]	45%	0.99	98%	61%	99%	48%	26%	0.59%	45%	29%
Alk Phos	0.93	[0.92, 0.94]	45%	0.95	92%	71%	93%	68%	37%	3.0%	42%	18%
Alk Phos	0.93	[0.92, 0.94]	45%	0.9	87%	78%	86%	79%	43%	6.3%	39%	11%
Alk Phos	0.93	[0.92, 0.94]	45%	0.8	79%	96%	69%	97%	53%	14%	31%	1.4%
Urea Nitrogen	0.93	[0.91, 0.94]	38%	0.99	98%	47%	99%	31%	19%	0.38%	38%	43%
Urea Nitrogen	0.93	[0.91, 0.94]	38%	0.95	92%	67%	90%	73%	45%	3.7%	34%	17%
Urea Nitrogen	0.93	[0.91, 0.93]	38%	0.9	89%	81%	83%	88%	54%	6.6%	31%	7.5%
Urea Nitrogen	0.93	[0.91, 0.94]	38%	0.8	79%	95%	58%	98%	61%	16%	22%	1.2%

eTable 8. Diagnostic Metrics for Common UCSF Components

Lab Test	Stanford -> Stanford	Stanford -> UCSF	Stanford -> UMich	UCSF -> Stanford	UCSF -> UCSF	UCSF -> UMich	UMich -> Stanford	UMich -> UCSF	UMich -> UMich

© 2019 Xu S et al. *JAMA Network Open*.

White Blood Cells	0.89 [0.88, 0.91]	0.88 [0.86, 0.89]	0.79 [0.77, 0.81]	0.87 [0.86, 0.89]	0.88 [0.87, 0.9]	0.81 [0.8, 0.83]	0.87 [0.86, 0.89]	0.88 [0.86, 0.89]	0.83 [0.81, 0.84]
Hemoglobin	0.93 [0.92, 0.94]	0.94 [0.93, 0.95]	0.89 [0.88, 0.91]	0.86 [0.84, 0.88]	0.9 [0.89, 0.92]	0.79 [0.77, 0.81]	0.92 [0.9, 0.93]	0.94 [0.93, 0.95]	0.9 [0.89, 0.91]
Platelet Count	0.91 [0.9, 0.92]	0.94 [0.93, 0.95]	0.91 [0.9, 0.93]	0.89 [0.88, 0.91]	0.95 [0.94, 0.96]	0.91 [0.89, 0.92]	0.89 [0.88, 0.91]	0.94 [0.93, 0.95]	0.92 [0.9, 0.93]
Sodium	0.87 [0.85, 0.88]	0.88 [0.86, 0.89]	0.91 [0.9, 0.93]	0.85 [0.84, 0.87]	0.89 [0.87, 0.9]	0.91 [0.89, 0.92]	0.86 [0.84, 0.88]	0.86 [0.84, 0.88]	0.91 [0.9, 0.93]
Potassium	0.76 [0.73, 0.79]	0.75 [0.73, 0.78]	0.67 [0.63, 0.7]	0.74 [0.71, 0.77]	0.77 [0.74, 0.79]	0.75 [0.72, 0.78]	0.73 [0.69, 0.76]	0.75 [0.72, 0.77]	0.76 [0.73, 0.79]
CO2	0.86 [0.84, 0.88]	0.8 [0.78, 0.82]	0.75 [0.71, 0.78]	0.8 [0.77, 0.82]	0.87 [0.86, 0.89]	0.85 [0.82, 0.87]	0.77 [0.74, 0.8]	0.82 [0.8, 0.84]	0.87 [0.84, 0.88]
Urea Nitrogen	0.95 [0.94, 0.96]	0.92 [0.91, 0.93]	0.9 [0.89, 0.92]	0.94 [0.93, 0.95]	0.92 [0.91, 0.93]	0.9 [0.89, 0.92]	0.93 [0.92, 0.94]	0.92 [0.91, 0.93]	0.92 [0.91, 0.93]
Creatinine	0.96 [0.96, 0.97]	0.91 [0.89, 0.92]	0.85 [0.83, 0.86]	0.94 [0.94, 0.95]	0.94 [0.93, 0.95]	0.88 [0.87, 0.9]	0.92 [0.91, 0.93]	0.88 [0.86, 0.89]	0.9 [0.88, 0.91]
Calcium	0.88 [0.87, 0.9]	0.86 [0.85, 0.88]	0.81 [0.79, 0.83]	0.87 [0.85, 0.88]	0.87 [0.86, 0.88]	0.85 [0.83, 0.86]	0.85 [0.83, 0.87]	0.86 [0.84, 0.87]	0.89 [0.88, 0.9]
Albumin	0.92 [0.91, 0.93]	0.88 [0.87, 0.9]	0.73 [0.7, 0.75]	0.84 [0.82, 0.86]	0.89 [0.88, 0.9]	0.74 [0.72, 0.76]	0.92 [0.9, 0.93]	0.89 [0.87, 0.9]	0.9 [0.89, 0.92]
Protein	0.91 [0.89, 0.92]	0.89 [0.88, 0.9]	0.87 [0.86, 0.88]	0.88 [0.87, 0.9]	0.89 [0.88, 0.9]	0.85 [0.83, 0.86]	0.89 [0.87, 0.9]	0.88 [0.86, 0.89]	0.9 [0.89, 0.91]
Alk Phos	0.94 [0.93, 0.95]	0.91 [0.9, 0.92]	0.89 [0.88, 0.91]	0.92 [0.91, 0.93]	0.93 [0.92, 0.94]	0.89 [0.87, 0.9]	0.92 [0.91, 0.93]	0.93 [0.92, 0.94]	0.92 [0.91, 0.93]
Total Bilirubin	0.96 [0.95, 0.97]	0.91 [0.89, 0.92]	0.91 [0.89, 0.93]	0.95 [0.94, 0.97]	0.93 [0.91, 0.94]	0.91 [0.89, 0.92]	0.96 [0.94, 0.97]	0.92 [0.9, 0.93]	0.92 [0.9, 0.93]
AST (SGOT)	0.92 [0.91, 0.93]	0.81 [0.8, 0.83]	0.86 [0.85, 0.87]	0.85 [0.83, 0.86]	0.77 [0.76, 0.79]	0.73 [0.71, 0.75]	0.88 [0.87, 0.9]	0.77 [0.75, 0.79]	0.86 [0.85, 0.87]
ALT (SGPT)	0.93 [0.92, 0.94]	0.86 [0.84, 0.87]	0.91 [0.9, 0.92]	0.92 [0.91, 0.93]	0.91 [0.9, 0.93]	0.88 [0.86, 0.89]	0.88 [0.87, 0.89]	0.84 [0.82, 0.86]	0.88 [0.87, 0.9]

eTable 9. Diagnostic Metrics for Common Components in Transferability Study

Lab Test	Medicare	Chargemaster
Magnesium	$8.27	$280.00
Prothrombin Time	$4.85	$190.00
Phosphorus	$5.85	$225.00
Partial Thromboplastin Time	$7.98	$240.00
Lactate	$11.87	$330.00
Calcium Ionized	$13.73	$399.00
Potassium	$5.68	$204.00
Troponin I	$12.47	$520.00
LDH Total	$6.71	$193.00
Heparin	$16.16	$466.00
Urinalysis	-	$196.00

© 2019 Xu S et al. *JAMA Network Open*.

Blood Culture (Aerobic & Anaerobic)	-	$499.00
Blood Culture (2 Aerobic)	-	$499.00
Sodium	$5.94	$219.00
Lidocaine	$18.14	$264.00
Hematocrit	$2.93	$217.00
Urine Culture	$9.96	$317.00
Urinalysis With Microscopic	$3.76	$196.00
Uric Acid	$5.58	$135.00
Hemoglobin A1c	$11.99	$145.00
Sepsis Protocol Lactate	$11.87	$330.00
iSTAT Troponin I	-	$520.00
Platelet Count	$5.53	$138.00
Lipase	$8.51	$213.00
Procalcitonin	$33.08	$318.00
Lactic Acid	$13.19	$330.00
Fibrinogen	$14.02	$285.00
Thyroid Stimulating Hormone	$20.75	$380.00
Creatine Kinase	$8.04	$254.00
C-Reactive Protein	$6.39	$179.00
NT-proBNP	$41.90	$821.00
Triglycerides	$7.09	$175.00
Ck	$14.26	$446.00
C.diff Toxin B Gene	-	$486.00
Osmolality	$8.16	$209.00
Respiratory Culture And Gram Stain	-	$226.00
Sedimentation Rate (ESR)	-	$75.00
Ferritin	$16.83	$375.00
Albumin	-	$138.00
Ammonia	-	$334.00
Specific Gravity	$3.28	$115.00
Fungal Culture	-	$309.00
Haptoglobin	-	$258.00
Anaerobic Culture	$11.66	$544.00
Fluid Culture And Gram Stain	-	$226.00
Cmv Dna Pcr Quant	-	$849.00
Blood Cult Central Line Catheter By Nurse	-	$499.00
T4	-	$246.00
Transferrin Saturation	$15.76	$292.50
Vitamin B12	$18.61	$281.00

© 2019 Xu S et al. *JAMA Network Open*.

Blood Cult - First Set	-	$499.00
Prealbumin	$18.01	$246.00
Osmolality	$8.42	$255.00
Digoxin	-	$389.00
Reticulocyte Count Automated	$4.93	$179.00
Cortisol	-	$374.00
Hepatitis B Antigen	$12.75	$220.00
Iron	-	$165.00
AFB Culture	-	$162.00
Calcium	-	$143.00
Biopsy/tissue With Gram Stain	-	$182.00
Afb Culture	-	$249.50
Pregnancy Test	$8.61	$102.00
Urea Nitrogen	-	$157.00
Gram Stain	-	$150.00
Teg	-	$747.00
Protein Total	$21.24	$229.00
Folic Acid	-	$274.00
Glucose	-	$250.00
Respiratory Culture	-	$302.00
CSF Culture And Gram Stain	-	$226.00
Occult Bld	-	$130.00
iSTAT Creatinine	$6.33	$179.00
iSTAT Cg4	-	$572.50
Anti-hiv	-	$280.00
Stool Culture	$11.66	$402.50

eTable 10. Medicare and Chargemaster Fees for Standalone Labs

© 2019 Xu S et al. *JAMA Network Open*.

lab	feature 1	score 1	feature 2	score 2	feature 3	score 3
**Hemoglobin A1c**	last_normality	0.437	Diabetes	0.154	HCT	0.054
**AFB Culture**	last_normality	0.639	LABAFBC	0.132	Birth	0.087
**Afb Culture**	AdmitDxDate	0.467	BP_Low_Diastolic	0.267	Pulse	0.2
**Albumin**	ALB	0.434	last_normality	0.094	Temp	0.086
**Anaerobic Culture**	last_normality	0.448	Temp	0.102	Pulse	0.078
**Vitamin B12**	ALB	0.136	TBIL	0.119	PLT	0.093
**Blood Culture (Aerobic & Anaerobic)**	Temp	0.178	Pulse	0.124	BP_High_Systolic	0.11
**Blood Culture (2 Aerobic)**	PLT	0.149	Pulse	0.119	last_normality	0.113
**Blood Cult - First Set**	WBC	0.252	Temp	0.168	K	0.128
**Blood Cult Central Line Catheter By Nurse**	Pulse	0.211	Temp	0.168	TBIL	0.1
**Urea Nitrogen**	BUN	0.408	LABBUN	0.14	CR	0.067
**Biopsy/tissue With Gram Stain**	last_normality	0.312	WBC	0.166	PLT	0.091
**Calcium**	CA	0.634	Pulse	0.083	last_normality	0.08
**Calcium Ionized**	CAION	0.344	last_normality	0.182	PHCAI	0.154
**C.difficile Toxin B Gene**	LABCDTPCR	0.295	Pulse	0.137	last_normality	0.089
**Creatine Kinase**	CK	0.411	last_normality	0.264	PHA	0.055
**Cmv Dna Pcr Quant**	CMVLOG	0.266	last_normality	0.212	CMVCP	0.084
**Cortisol**	K	0.187	LAC	0.121	CO2	0.117
**C-Reactive Protein**	CRP	0.217	Temp	0.161	last_normality	0.152
**CSF Culture And Gram Stain**	WBC	0.173	Pulse	0.14	Temp	0.139
**Glucose**	GLUCSF	0.393	last_normality	0.109	NA	0.068
**Protein Total**	TPCSF	0.191	Pulse	0.113	BP_High_Systolic	0.091
**Digoxin**	last_normality	0.759	DIG	0.188	Pulse	0.024
**Sedimentation Rate (ESR)**	HCT	0.269	last_normality	0.134	ALB	0.12
**Fungal Culture**	last_normality	0.152	Resp	0.116	Pulse	0.111
**Iron Total**	Temp	0.158	Male	0.125	PLT	0.121
**Ferritin**	last_normality	0.345	ALB	0.175	Temp	0.072
**Fibrinogen**	FIBRINOGEN	0.463	last_normality	0.237	PLT	0.046
**Fluid Culture And Gram Stain**	Temp	0.175	SurgerySpecialty	0.134	Pulse	0.106
**Folic Acid**	Birth	0.195	CA	0.129	BP_High_Systolic	0.103

© 2019 Xu S et al. *JAMA Network Open*.

**T4 Free**	BP_High_Systolic	0.155	last_normality	0.124	Temp	0.097
**Gram Stain**	BP_High_Systolic	0.116	BP_Low_Diastolic	0.093	Resp	0.091
**Haptoglobin**	last_normality	0.547	TBIL	0.08	PLT	0.058
**Hepatitis B Antigen**	last_normality	0.302	AdmitDxDate	0.284	order_time	0.067
**Hematocrit**	HCT	0.577	last_normality	0.114	Resp	0.061
**Heparin**	last_normality	0.431	HEPAR	0.179	Resp	0.078
**Anti-hiv**	PLT	0.255	HCT	0.196	Temp	0.101
**Potassium**	K	0.678	last_normality	0.085	HCT	0.082
**Lactic Acid**	LAC	0.504	last_normality	0.263	PHA	0.044
**Lactate**	last_normality	0.707	LACWBL	0.138	Pulse	0.027
**LDH Total**	last_normality	0.773	LDH	0.168	WBC	0.01
**Lidocaine**	LIDO	0.329	last_normality	0.315	BP_Low_Diastolic	0.078
**Lipase**	last_normality	0.66	Pulse	0.047	LIPASE	0.045
**Ck Mb (mass)**	last_normality	0.31	CKMBRI	0.124	CKMB	0.109
**Magnesium**	MG	0.471	last_normality	0.235	Resp	0.044
**Sodium**	last_normality	0.659	NA	0.295	Pulse	0.009
**Ammonia**	NH3	0.362	last_normality	0.202	PLT	0.076
**Nt - Probnp**	last_normality	0.318	Birth	0.204	BUN	0.094
**Osmolality**	NA	0.338	last_normality	0.303	OSMOL	0.276
**Prealbumin**	PREALBUMIN	0.591	ALB	0.064	HCT	0.056
**iSTAT Cg4**	Pulse	0.273	BP_Low_Diastolic	0.176	Temp	0.129
**iSTAT Creatinine**	CR	0.571	Birth	0.172	BP_High_Systolic	0.166
**iSTAT Troponin I**	Comorbidity.MI	0.376	last_normality	0.161	TNI	0.155
**Phosphorus**	last_normality	0.472	PHOS	0.153	CR	0.066
**Platelet Count**	PLT	0.719	HCT	0.061	Temp	0.031
**Procalcitonin**	CR	0.19	last_normality	0.139	PROCTL	0.101
**Prothrombin Time**	PT	0.348	INR	0.262	last_normality	0.15
**Teg**	PO2V	0.237	PCO2V	0.201	PO2A	0.156
**Partial Thromboplastin Time**	last_normality	0.756	PTT	0.077	Pulse	0.032
**Respiratory Culture**	last_normality	0.351	Urine	0.161	PCO2A	0.117
**Respiratory Culture And Gram Stain**	last_normality	0.359	Glasgow Coma Scale Score	0.131	Resp	0.111

© 2019 Xu S et al. *JAMA Network Open*.

**Reticulocyte Count Automated**	last_normality	0.487	HCT	0.254	TBIL	0.076
**Sepsis Protocol Lactate**	LACWBL	0.3	last_normality	0.125	Temp	0.103
**Stool Culture**	BP_Low_Diastolic	0.161	Temp	0.108	Pulse	0.092
**Occult Bld**	K	0.476	order_time	0.256	WBC	0.051
**Troponin I**	TNI	0.442	last_normality	0.29	Pulse	0.045
**Transferrin Saturation**	ALB	0.241	HCT	0.14	PLT	0.125
**Triglycerides**	TGL	0.603	last_normality	0.244	Birth	0.017
**Thyroid Stimulating Hormone**	last_normality	0.181	BP_High_Systolic	0.143	BP_Low_Diastolic	0.131
**Osmolality**	K	0.254	CO2	0.189	BUN	0.187
**Urinalysis With Microscopic**	Pulse	0.242	LABUA	0.142	AdmitDxDate	0.136
**Urinalysis**	Pulse	0.128	BP_High_Systolic	0.125	Temp	0.095
**Pregnancy Test**	AdmitDxDate	0.177	Pulse	0.167	BP_Low_Diastolic	0.155
**Uric Acid**	URIC	0.26	last_normality	0.238	CR	0.161
**Urine Culture**	Male	0.241	last_normality	0.142	Temp	0.131
**Specific Gravity**	SPG	0.2	HCT	0.2	Temp	0.2

eTable 11: Top 3 Important Features for Stanford Standalone Labs

lab	feature 1	score 1	feature 2	score 2	feature 3	score 3
**White Blood Cells**	WBC	0.558	last_normality	0.244	PLT	0.065
**Hemoglobin**	HCT	0.287	HGB	0.217	last_normality	0.181
**Platelet Count**	last_normality	0.665	PLT	0.238	Pulse	0.014
**Sodium**	last_normality	0.787	NA	0.184	Resp	0.009
**Potassium**	last_normality	0.576	K	0.271	Pulse	0.067
**Creatinine**	last_normality	0.87	CR	0.1	Birth	0.005
**Urea Nitrogen**	last_normality	0.809	BUN	0.089	CR	0.017
**CO2**	CO2	0.539	last_normality	0.141	Pulse	0.053
**Calcium**	CA	0.532	last_normality	0.246	Pulse	0.042
**Protein**	TP	0.677	last_normality	0.117	CA	0.033
**Albumin**	ALB	0.459	last_normality	0.208	Pulse	0.107

© 2019 Xu S et al. *JAMA Network Open*.

**Alk Phos**	ALKP	0.672	last_normality	0.235	AdmitDxDate	0.016
**Total Bilirubin**	TBIL	0.62	last_normality	0.307	PLT	0.019
**AST (SGOT)**	AST	0.642	last_normality	0.217	Resp	0.028
**ALT (SGPT)**	last_normality	0.848	ALT	0.115	ALB	0.008

eTable 12. Top 3 Important Features for Common Stanford Components

lab	feature 1	score 1	feature 2	score 2	feature 3	score 3
**Magnesium**	last_normality	0.605	MAG	0.286	AdmitDxDate	0.033
**Phosphorus**	PHOS	0.53	last_normality	0.154	CREAT	0.088
**Hemoglobin A1c**	AdmitDxDate	0.171	order_time	0.141	Birth	0.099
**Uric Acid**	last_normality	0.643	URIC	0.241	HCT	0.032
**Albumin**	ALB	0.365	HCT	0.157	CAL	0.109
**Thyroid Stimulating Hormone**	AdmitDxDate	0.173	order_time	0.139	Birth	0.122
**Troponin I**	TROP	0.683	last_normality	0.151	AdmitDxDate	0.035
**Potassium**	POT	0.364	Birth	0.116	AdmitDxDate	0.11
**Sodium**	SOD	0.714	last_normality	0.082	POT	0.057
**Calcium**	CAL	0.505	WBC	0.17	CREAT	0.105

eTable 13. Top 3 Important Features for Top UMich Standalone Labs

lab	feature 1	score 1	feature 2	score 2	feature 3	score 3
**White Blood Cells**	last_normality	0.62	WBC	0.217	AdmitDxDate	0.048
**Hemoglobin**	HGB	0.39	last_normality	0.338	HCT	0.209
**Platelet Count**	last_normality	0.836	PLT	0.119	Birth	0.016
**Sodium**	last_normality	0.837	SOD	0.11	AdmitDxDate	0.035
**Potassium**	POT	0.667	last_normality	0.16	CREAT	0.063
**Creatinine**	last_normality	0.881	CREAT	0.071	AdmitDxDate	0.017

© 2019 Xu S et al. *JAMA Network Open*.

**Total Bilirubin**	TBIL	0.475	last_normality	0.211	PLT	0.109
**CO2**	last_normality	0.755	CO2	0.174	AdmitDxDate	0.026
**AST (SGOT)**	last_normality	0.753	AST	0.176	Birth	0.026
**ALT (SGPT)**	last_normality	0.853	ALT	0.118	Birth	0.011
**Albumin**	last_normality	0.802	ALB	0.074	Birth	0.054
**Calcium**	CAL	0.622	last_normality	0.266	AdmitDxDate	0.021
**Protein**	PROT	0.446	last_normality	0.222	HCT	0.059
**Alk Phos**	last_normality	0.775	ALK	0.113	Birth	0.044
**Urea Nitrogen**	last_normality	0.79	UN	0.116	Birth	0.034

eTable 14. Top 3 Important Features for Common UMich Components

**lab**	**feature 1**	**score 1**	**feature 2**	**score 2**	**feature 3**	**score 3**
**Magnesium**	last_normality	0.585	MG	0.297	LABMGN	0.044
**Phosphorus**	PO4	0.483	last_normality	0.169	CREAT	0.098
**Prothrombin Time**	PT	0.46	INR	0.221	last_normality	0.152
**Partial Thromboplastin Time**	PTT	0.593	last_normality	0.127	LABPTT	0.091
**Alkaline Phosphatase**	ALKP	0.643	last_normality	0.183	Alkaline Phosphatase	0.038
**Sodium**	NA	0.941	Pulse	0.015	last_normality	0.012
**Potassium**	K	0.558	last_normality	0.168	LABK	0.067
**Troponin I**	TRPI	0.302	last_normality	0.25	LABTNI	0.222
**Lactate Dehydrogenase**	LD	0.595	last_normality	0.193	LABLDH	0.1
**Calcium, Ionized**	CAI	0.529	last_normality	0.191	CA	0.172
**Uric Acid**	URIC	0.629	last_normality	0.217	PLT	0.02
**Albumin**	ALB	0.358	CA	0.187	HCT	0.107
**Thyroid Stimulating Hormone**	last_normality	0.247	HCT	0.205	Temp	0.117

eTable 15. Top 3 Important Features for Top UCSF Standalone Labs

© 2019 Xu S et al. *JAMA Network Open*.

lab	feature 1	score 1	feature 2	score 2	feature 3	score 3
**White Blood Cells**	last_normality	0.719	WBC	0.216	Pulse	0.024
**Hemoglobin**	last_normality	0.767	HGB	0.128	DBP	0.025
**Platelet Count**	last_normality	0.886	PLT	0.091	SBP	0.007
**Sodium**	last_normality	0.755	NA	0.191	CREAT	0.012
**Potassium**	K	0.442	last_normality	0.118	SBP	0.072
**Creatinine**	last_normality	0.836	CREAT	0.088	Comorbidity.RenalDisease	0.034
**Total Bilirubin**	last_normality	0.791	TBILI	0.138	SBP	0.014
**CO2**	CO2	0.684	last_normality	0.218	HCT	0.025
**AST (SGOT)**	last_normality	0.715	AST	0.196	Pulse	0.019
**ALT (SGPT)**	ALT	0.587	last_normality	0.176	Pulse	0.074
**Albumin**	ALB	0.502	last_normality	0.211	CA	0.078
**Calcium**	CA	0.734	last_normality	0.125	Pulse	0.031
**Protein**	TP	0.656	last_normality	0.137	DBP	0.048
**Alk Phos**	last_normality	0.765	ALKP	0.175	SBP	0.01
**Urea Nitrogen**	last_normality	0.745	BUN	0.139	Comorbidity.RenalDisease	0.018

eTable 16. Top 3 Important Features for Common UCSF Components

© 2019 Xu S et al. *JAMA Network Open*.

eMethods. Technical Details of Machine Learning Algorithm

1.Recursive feature elimination with cross validation (using a RandomForest estimator)

We apply recursive feature elimination with cross validation (RFECV) to select top 5% relevant features for prediction as implemented by scikit-learn (http://scikit-learn.org/). This process uses all features in the development set to train a random forest prediction model, identifying which features appear the least important towards the predicted outcome as assessed by a Gini entropy score. We removed those least relevant features and repeated this process recursively, until there were only 5% of the total original features left.

2. Hyperparameter tuning for machine learning algorithms

Each of the eight applied machine learning algorithms includes additional "hyperparameters" that can tune the performance of the algorithms. For example, penalized logistic regression includes a regularization penalty that specifies how much the algorithm should balance using more information vs. developing the simplest model that uses the fewest number of features. Similarly, random forest models have a maximum tree depth hyperparameter that constrains how many features are considered when building decision trees, to balance the bias-variance tradeoff that can impact the generalizability of model accuracy. We systematically tested all models across a range of plausible hyperparameter values to identify the most effective choices (eTable 1).

3. Applying locally trained model to a remote dataset

We apply pre-trained models to data of a remote site by manually mapping identical columns. For example, when applying Stanford model to the UMich dataset, we use a "Stanford template" to process the full UMich feature matrix with 603 columns. The template includes 43 columns imputed and selected (by RFECV) from Stanford training set. The same set of 43 features were then select from the UMich matrix and imputed accordingly if they exist. If instead, the UMich dataset does not have a corresponding feature (e.g. patient vitals), we will create a dummy column with the same feature name but filled with the corresponding constant imputation value. The generated UMich feature matrix has then "Stanford-like" format and is ready to be fed into Stanford-trained model.

© 2019 Xu S et al. *JAMA Network Open*.
